# Research Progress on Nanopolymer Composites in Civil Engineering

**DOI:** 10.3390/nano16040267

**Published:** 2026-02-18

**Authors:** Tingting Gao, Yan Zhao, Yanan Niu, Xi Cao

**Affiliations:** 1 Shandong College of Electronic Technology, Jinan 250200, China; 2 School of Civil Engineering and Architecture, Wuyi University, Wuyishan 354300, China; 3 College of Materials Science and Engineering, Jiamusi University, Jiamusi 154007, China; 4 School of Information Science and Technology, Beijing University of Chemical Technology, Beijing 100029, China

**Keywords:** nanopolymer composites, civil engineering, weather resistance, toughness optimization, interfacial modification, application progress

## Abstract

Civil engineering infrastructure suffers material degradation, shortened service life and high maintenance costs under harsh environments and natural aging, threatening public safety. Nanopolymer composites, featuring designable microstructures and excellent macroscopic properties, provide a revolutionary solution to improve the weather resistance and toughness of civil engineering materials. This paper systematically clarifies the modification mechanisms of nanocomposites, focusing on nanofiller–polymer matrix interfacial interactions (physical adsorption, chemical bonding) and their synergistic effects in enhancing environmental aging resistance (UV, corrosion, freeze–thaw) and mechanical performance (toughening, strengthening, dynamic load resistance). It summarizes the latest applications in nanomodified protective coatings, sealing/bonding materials and composite structural components, revealing the inherent “structure-property-application” relationships. Furthermore, this paper addresses core large-scale application challenges, including technical bottlenecks, performance evaluation limitations and economic/environmental barriers. Finally, future research directions are proposed, covering multifunctional intelligent materials, green development, interdisciplinary computational methods and standardized systems. This review offers an integrated perspective, providing theoretical guidance and practical references for advancing durable, resilient and sustainable civil engineering.

## 1. Introduction

Civil engineering infrastructure such as bridges, buildings, and offshore platforms endures prolonged exposure to the combined effects of severe environmental loads and natural aging factors. Ultraviolet radiation, moisture penetration, freeze–thaw cycles, chemical corrosion (e.g., from chloride ions and sulfates), and increasingly frequent extreme weather events continuously threaten the structural integrity and service life of conventional materials [1]. The resulting performance degradation—primarily manifested as concrete carbonation, reinforcement corrosion, polymer coating chalking, and interfacial delamination in composite materials—not only incurs substantial economic maintenance costs but also poses potential risks to public safety [2,3]. Therefore, developing a new generation of high-performance, highly durable engineering materials to proactively address these challenges has become an urgent scientific need and technological objective in the field of civil engineering.

Against this backdrop, nanopolymer composites stand out as a revolutionary material solution due to their designable microstructure and exceptional macroscopic properties. By incorporating low concentrations of nanoscale fillers—such as carbon nanotubes, graphene, nano-silica, or nanoclay—into polymer matrices like epoxy, polyurethane, or vinyl ester, material properties can be fundamentally optimized at both molecular and nanoscale levels [4]. Compared to traditional micron-scale composites, nanocomposites exhibit two core advantages: First, these have a pronounced interfacial effect. The enormous specific surface area of nanoparticle fillers enables strong physical or chemical interactions with the polymer matrix, creating highly efficient hubs for stress transfer and energy dissipation. Second, these show multifunctional integration potential. Different types of nanoparticle fillers can individually or synergistically impart functional properties beyond structural reinforcement—such as electrical conductivity, thermal conductivity, barrier properties, and aging resistance [5].

In civil engineering applications, this nanoscale modification strategy primarily focuses on breakthroughs in two core properties: weather resistance and toughness. Enhanced weather resistance enables materials to withstand environmental erosion for longer periods, thereby extending structural lifespan. Optimized toughness directly relates to a material’s crack resistance and fracture ductility under overload, impact, or fatigue loading, serving as a critical safeguard for structural safety [6,7].

Although numerous studies have focused on specific nanoparticle fillers or individual applications [8,9], there remains a lack of a systematic review that treats nanopolymer composites as a unified platform. Such a review should horizontally integrate three key civil engineering material domains—protective coatings, sealants, and fiber-reinforced polymer (FRP) composite components—while vertically delving into the common and specific mechanisms governing their weatherability and toughness optimization.

Recent reviews have primarily focused on specific filler–matrix systems such as cementitious nanocomposites and epoxy/graphene, or cross-disciplinary general applications. This review innovatively establishes an integrated “structure-property-application” framework centered on interface engineering. This study achieves breakthroughs through the following aspects: (1) horizontally integrating three major civil engineering material domains—protective coatings, sealants, and FRP components—while vertically elucidating the multiscale mechanisms governing weatherability and toughness optimization; (2) revealing the intrinsic “dimension-mechanism-performance” relationship through dimension-oriented functional classification; and (3) establishing an application-oriented roadmap bridging laboratory research and engineering practice. This integrated perspective provides both mechanistic foundations for researchers and practical guidelines for engineers. A comparison with recent related reviews is presented in Table 1 below.

Given this, this review will first systematically elucidate the fundamental scientific mechanisms of nanomodification, particularly how interfacial interactions synergistically enhance materials’ resistance to environmental aging and fracture toughness. Subsequently, we will systematically present the latest research advances in nanocomposites across three major application domains, revealing the intrinsic relationship between “structure-property-application” through comparative analysis. Furthermore, we will directly address the critical challenges currently facing this field in terms of large-scale production, long-term performance evaluation, cost-effectiveness, and environmental health and safety. Building upon this foundation, we will propose a clear roadmap for future interdisciplinary research. Finally, this paper will summarize the core value and implementation pathways of nanopolymer composites in propelling civil engineering toward a more durable, resilient, and sustainable future.

The innovation of this review lies in providing an integrated perspective that spans materials, performance characteristics, and applications. It not only clarifies the technological development trajectory and core scientific challenges for researchers but also offers engineers and industry professionals a mechanism-based rationale for selecting and implementing these advanced materials—translating them from the laboratory to practical engineering applications.

## 2. Mechanisms of Nanoscale Modification and Key Performance Enhancements

### 2.1. Nanofiller–Polymer Interface Interactions

The interface region between nanofillers and the polymer matrix is a decisive factor in the performance of composite materials. This interfacial interaction can be primarily categorized into three levels: physical interactions [11], chemical bonding [12], and the resulting interfacial stress transfer mechanism [13].

**Figure 1:**
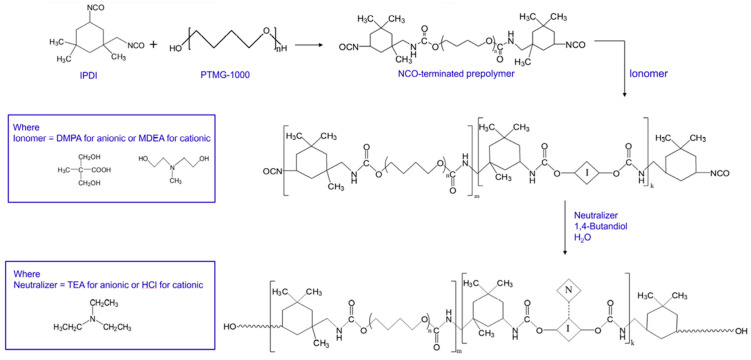
Polymerization process for waterborne polyurethanes [17].

First, the enormous specific surface area of nanofillers (such as carbon nanotubes and graphene flakes) enables them to tightly adsorb onto polymer chains through physical interactions including van der Waals forces [14], mechanical interlocking [15] and hydrogen bonding [16]. For instance, Kim et al. [17] focused on the performance optimization of waterborne polyurethane (WPU) nanocomposites with graphene oxide (GO) and multiwalled carbon nanotubes (MWCNT). To address the poor compatibility between anionic WPU and GO due to like-charge repulsion, they enhanced compatibility through ionic interactions by either modifying nanofillers (NFs) with cationic surfactants or preparing cationic WPU (as shown in Figure 1). Research indicates that when WPU and NFs possess opposite ionic charges, the composite exhibits significantly improved dispersion stability, mechanical properties, and glass transition temperature. Furthermore, the ionic monomer content influences the optimal NF loading, providing an effective strategy for regulating the performance of WPU-based nanocomposites. Mahendia et al. [18] employed a modified Hammer method to synthesize reduced graphene oxide (RGO) and prepared RGO/polyvinyl alcohol (PVA) composites via solution casting. Characterization using UV-Vis, Raman, FTIR, and DSC confirmed strong hydrogen bonding interactions between RGO and PVA. Using two-dimensional mapping with thermally sensitive FTIR to determine the glass transition temperature (Tg), it was found that 0.5 wt.% RGO doping increased PVA’s Tg from 78 °C to 92 °C, consistent with the DSC results, providing an effective means for regulating the thermal properties of composites. Furthermore, more decisive chemical bonding can be achieved through surface functionalization of the filler. Covalent modification of carbon nanotubes with amino (-NH_2_) or epoxy groups enables their direct participation in the crosslinking network during epoxy resin curing, forming strong covalent bonds. Khan et al. [19] prepared two modified fillers, MDA-B-GO and BDM-B-GO, by covalently grafting amine-terminated oligomeric imide chains onto the surface of graphene oxide (GO). These fillers were then combined with epoxy resin to form nanocomposites. The oligomeric imide chains suppressed GO agglomeration, while their amine groups formed covalent bonds with epoxy, enhancing the composites’ thermal conductivity by 52% and 56%, respectively. This significantly improved mechanical properties and thermal stability, providing an effective modification strategy for high-performance epoxy-based composites. Seraji et al. [20] modified aerogel-spun carbon nanotube (CNT) fibers through a two-step post-treatment process involving acid treatment and epoxy impregnation. Acid treatment reduced impurities in CNT fibers and introduced carboxyl groups, but also generated surface defects; epoxy impregnation filled fiber pores and enhanced interfacial interactions. Composites prepared using a 20 wt.% epoxy bath exhibited optimal properties, with significantly enhanced tensile strength and modulus compared to the original fibers. Acid treatment increased strength but reduced modulus, providing structural design insights for high-performance CNT fiber composites. Meanwhile, inorganic nanoparticles play a pivotal role in composite materials such as geopolymers. For instance, allophane nanotubes can uniformly disperse beeswax particles through Pickering emulsion stabilization. Even at low loading levels, they significantly enhance the material’s flexural strength and thermal storage capacity while regulating its pore structure. Their unique morphology and interfacial interactions optimize the microstructure of composites, providing crucial support for the development of eco-friendly functional building materials [21]. Lisuzzo et al. [22] investigated elohite nanotubes as a representative inorganic nanofiller. Leveraging their unique tubular structure and excellent dispersibility, these nanotubes demonstrate significant effects in composites such as bioplastics and geopolymers. Even at low loading levels (approximately 10 wt%), they enhance material thermal stability, mechanical properties, and heat storage capacity by optimizing microstructure. However, excessive loading tends to cause agglomeration and performance degradation. This research provides crucial support for developing multifunctional eco-friendly composites. Zhu et al. [23] investigated inorganic particles (e.g., OMMT, HB-Fe_3_O_4_, SiO_2_) as key nanofillers in inorganic microcapsule (IM) preparation, where they serve dual roles in template stabilization and functionalization. Through freeze-assisted interfacial reactions, they establish stable water–water interfaces, regulate shell structures and properties, confer magnetic responsiveness and biodegradability to IMs, and enable inhibitor encapsulation. This approach delivers highly efficient functional material solutions for civil engineering, catalysis, and other fields. Research by Ren et al. [24] demonstrates that inorganic particles—such as hematite, magnesite, OMMT, and Fe_3_O_4_-serve as functional key components when used as nanofillers in materials like geopolymers and inorganic microcapsules. These materials can modulate setting times, pore structures, and mechanical properties, enhance durability against acid resistance and corrosion, and impart magnetic responsiveness through interfacial reactions, providing core support for the precise design of multifunctional composites. In summary, the interfacial regulation strategy combining physical interactions and covalent bonding specifically addresses dispersion and compatibility challenges in nanofillers, significantly optimizing multiple composite properties. This provides a core conceptual framework and technical support for designing and applying high-performance polymer–matrix nanocomposites.

The aforementioned strong interfacial interactions collectively form an efficient stress transfer and energy dissipation mechanism. When the composite material is loaded, the load is effectively transferred from the relatively flexible polymer matrix to the high-modulus nanofillers through the robust interfaces. At the crack propagation front, high-strength nanofillers (such as carbon nanotubes) can bridge the crack edges, consuming substantial energy through a “pull-out” process; simultaneously, nanoparticles (e.g., rubber particles or hollow silica nanoparticles) act as stress concentration points, inducing large-scale shear yielding or plastic deformation in the matrix, thereby achieving significant toughening effects [25,26]. Therefore, precise design and control of the interface region form the physicochemical foundation for achieving a leap in the performance of nanocomposites.

The strength of these interfacial interactions directly determines stress transfer efficiency and energy dissipation capacity, ultimately quantifying as enhanced macroscopic performance. Although physically adsorbed interactions (such as van der Waals forces and hydrogen bonds) are weaker than chemical bonds, their cumulative effect becomes highly significant when dispersed at the nanoscale. For instance, a strong hydrogen bond network can effectively restrict the thermal motion of polymer segments, quantitatively manifested as a significant increase in glass transition temperature (Tg). Research by Mahendia et al. [18] demonstrates that adding just 0.5 wt.% reduced graphene oxide (RGO) can elevate the composite material’s Tg from 78 °C to 92 °C (ΔTg = +14 °C) through strong hydrogen bonding interactions formed between RGO and PVA. This change was verified through two-dimensional mapping using temperature-dependent Fourier transform infrared spectroscopy (FTIR), providing direct spectroscopic evidence and quantitative metrics for the strength of interfacial interactions.

More decisive chemical bonds, such as covalent bonds, establish more efficient stress transfer pathways. Their macroscopic reinforcement efficiency can be preliminarily explained by the classical shear-lag model, which relates the strength enhancement of composites to parameters like interfacial shear strength (τ) and filler aspect ratio. Strong covalent interfaces imply higher τ values, enabling near-ideal load transfer from low-modulus polymer matrices to high-modulus nanofillers (e.g., carbon nanotubes with modulus ~1 TPa). Khan et al. [19] provide compelling evidence: covalent grafting of amine-terminated oligomeric imide chains onto graphene oxide (GO) surfaces resulted in epoxy nanocomposites exhibiting 52% to 56% enhanced thermal conductivity. This dramatic performance improvement stems from the covalent bond interface significantly reducing interfacial thermal resistance due to phonon scattering, quantitatively demonstrating how “strong interfaces” enable “high-efficiency energy (heat) transfer.”

In summary, the type of filler (e.g., 0D, 1D, 2D) and its surface chemistry determine the interaction mechanisms with the matrix, while the dispersion quality of the filler is both a prerequisite and a key controlling factor for realizing these mechanisms. Even when employing high-performance nanofillers (e.g., graphene, carbon nanotubes), agglomeration due to poor dispersion not only fails to deliver reinforcement benefits but may also create stress concentration points and defect sources, significantly compromising the mechanical consistency and environmental durability of composites. Therefore, in nanocomposite design, controlling dispersion quality often takes precedence over simply increasing filler loading, serving as the foundation for achieving reproducible performance and engineering applicability.

### 2.2. Mechanism of Enhanced Weather Resistance

Polymer-based composites subjected to outdoor service continuously endure ultraviolet radiation, temperature and humidity fluctuations, and corrosion from aggressive media, making them prone to molecular chain degradation, surface cracking, and performance deterioration. Through precise control of nanofillers, multiple protective mechanisms can be established to synergistically enhance weather resistance at the levels of physical shielding, chemical inhibition, and structural stabilization. The core of this approach still relies on the efficient interaction and functional synergy at the filler–matrix interface. This section focuses on how nanofillers enhance the long-term stability of composite materials under environmental erosion—such as ultraviolet radiation, moisture/corrosive media, and freeze–thaw cycles—through mechanisms including physical shielding, chemical stabilization, and structural regulation. Although crack inhibition is also involved during freeze–thaw cycles, this section emphasizes weathering resistance enhancement mechanisms from the perspective of environmental erosion resistance, such as ice crystal expansion suppression, pore filling, and interfacial thermal stress regulation. The following sections elaborate on resistance mechanisms against three typical environmental erosion types.

#### 2.2.1. Resistance to UV Aging

High-energy photons from ultraviolet radiation can trigger photo-oxidative degradation of polymer molecular chains [27], leading to material yellowing, embrittlement, and diminished mechanical properties [28]. Semiconductor nanoparticles such as TiO_2_ and ZnO leverage their unique optical properties and interfacial control capabilities to establish a triple anti-aging mechanism of “scattering-absorption-inhibition” [29], whose efficacy is closely related to particle size, dispersion, and interfacial bonding strength [30].

From a mechanism perspective, the protective efficacy of nanoparticles exhibits particle size dependence: micron-sized particles primarily rely on high refractive indices to achieve UV reflection and scattering, with TiO_2_ demonstrating superior UVB shielding [31], and ZnO excelling at scattering UVA [32]. When particle size shrinks to the nanoscale (<100 nm), quantum confinement effects become prominent [33], shifting the protective mechanism toward UV absorption-when photon energy exceeds the particle’s bandgap (TiO_2_ at 3.2 eV corresponding to 387 nm, ZnO at 3.3 eV corresponding to 376 nm), valence band electrons are excited to the conduction band, forming electron-hole pairs that convert light energy into harmless thermal or chemical energy [34,35]. For example, studies by Miklecic et al. indicate that ZnO nanoparticles can shorten coating flow time and increase pH values; both types of nanoparticles enhance coating color stability by reducing ultraviolet light transmittance, with higher TiO_2_ concentrations yielding better results but causing coating haze. Additionally, TiO_2_ nanoparticles significantly increase the glass transition temperature (Tg), while ZnO nanoparticles reduce coating elongation and increase brittleness. Pigments can enhance coating durability without excessively compromising transparency [36].

The efficacy of these protective mechanisms can be characterized through quantitative changes in optical properties. The ultraviolet shielding efficiency of nanoparticles—such as reduced transmittance at specific wavelengths—does not follow a simple linear relationship with loading. Instead, it depends on their dispersion state within the matrix, particle size distribution, and the degree of refractive index matching with the matrix after surface modification. For instance, Miklecic et al. [36] systematically quantified the impact of TiO_2_ and ZnO nanoparticles on the optical properties of water-based polyacrylate coatings. Their findings revealed that nanoparticle incorporation significantly reduced transmittance in the UV region, with TiO_2_ exhibiting superior shielding effects at higher concentrations. However, this improvement may be accompanied by increased haze in the coating due to enhanced light scattering. These quantitative measurements of transmittance, color difference, and haze directly link the abstract “shielding mechanism” to engineered coating performance metrics.

**Figure 2:**
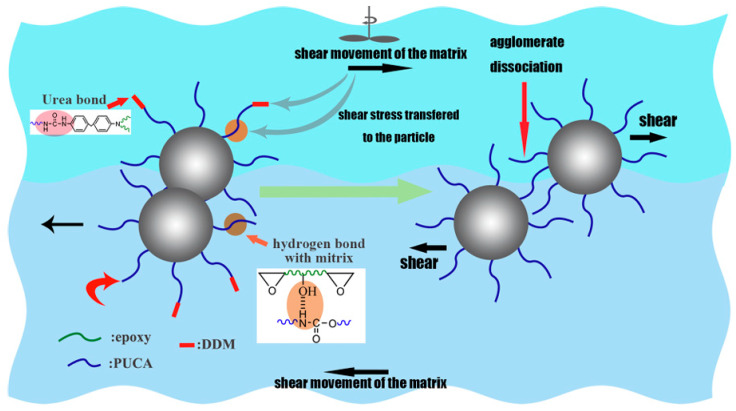
The dispersion promotion mechanism of the PUCA molecule grafted on the SiO_2_ NPs during the stirring process [38].

Interface regulation is critical for protective stability: unmodified nanoparticles readily agglomerate, creating protective blind spots. Improving their compatibility with the substrate through silane coupling agent modification or polymer grafting enables the formation of a uniform optical protective network [37]. Yuan et al. [38] synthesized a coupling agent (PUCA) containing isocyanate-terminated polyurethane chains and grafted it onto the surface of SiO_2_ nanoparticles via the sol-gel method, as shown in Figure 2 below. The results indicate that PUCA-SiO_2_ exhibits more uniform dispersion in epoxy resin, significantly enhancing the tensile properties of the composite material. The glass transition temperature (Tg) increased by 8.9 °C and 5.8 °C compared to pristine SiO_2_ and IPTS-SiO_2_, respectively. This confirms that the modification strategy effectively optimizes nanoparticle dispersion and composite material performance.

#### 2.2.2. Water Resistance/Corrosion Resistance

The permeation of water, oxygen, and corrosive ions (Cl^−^, SO_4_^2−^) constitutes the primary cause of composite hydrolysis and metal substrate corrosion [39]. Two-dimensional sheet-like fillers such as graphene and nanoclay can enhance corrosion resistance by synergistically forming a “maze-like” barrier structure [40] with electrochemical protection [41]. The effectiveness of this approach depends on the degree of oriented packing of the fillers and the integrity of the interfacial bonding.

The barrier performance of two-dimensional lamellar fillers can be conceptualized and semi-quantitatively described using the tortuosity model. This model indicates that when lamellar fillers are oriented parallel to the substrate within the coating, the diffusion paths for corrosion media (H_2_O, O_2_, Cl^−^) within the coating are significantly elongated and tortuously altered. The effective diffusion coefficient (Deff) is closely correlated with the filler’s aspect ratio (α) and volume fraction (φ), typically following a relationship of the form Deff ∝ D0/(1 + αφ). Thus, even at low loading levels, graphene or nanoclay with high aspect ratios can substantially reduce medium permeation rates. This barrier effect manifests macroscopically as a dramatic increase in coating impedance. Liu et al. [42] provided a typical example: Using a quinacridone (QA)-based covalent–noncovalent bridging modification strategy, an epoxy coating containing only 0.2 wt.% graphene maintained a stable low-frequency impedance modulus exceeding 10^10^ Ω·cm^2^ after prolonged immersion in harsh acidic brine (pH = 1, 3.5% NaCl). This value is several orders of magnitude higher than that of unmodified pure epoxy coatings, providing intuitive and quantitative evidence of the optimized physical barrier formed by the nanolayer interface and its exceptional ability to inhibit electrochemical corrosion processes.

**Figure 3:**
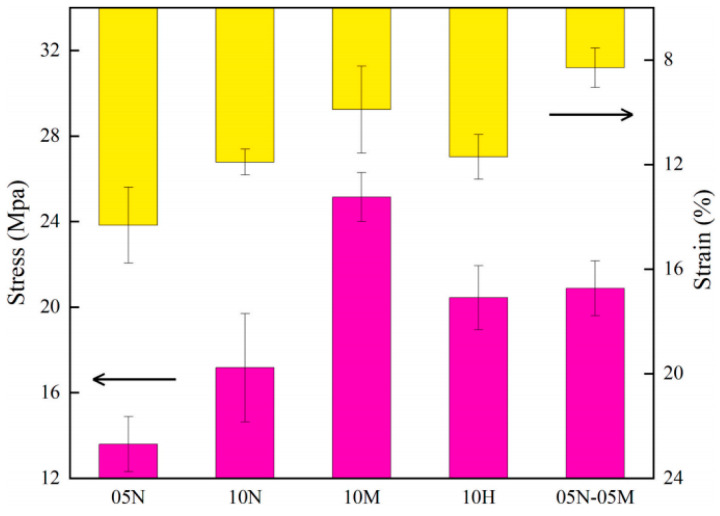
Fracture stress and strain of QA modified Gr/epoxy coatings, the left arrow corresponds to the left y-axis, which represents stress (MPa), while the right arrow corresponds to the right y-axis, which represents strain (%) [42].

The path lengthening effect stems from the high aspect ratio of two-dimensional fillers: when graphene or nanoclay is oriented parallel to the material surface, it can extend the diffusion path of the permeating medium by several to dozens of times, significantly reducing the permeation rate [43]. Liu et al. [42] employed a quinacridone (QA)-based covalent–noncovalent bridging modification strategy to prepare graphene/epoxy coatings. The composite system containing 0.2 wt% graphene with 0.5 wt% unmodified epoxy+0.5 wt% QA-modified epoxy exhibited the optimal performance. The fracture stress and strain of QA-modified graphite/epoxy coatings are shown in Figure 3. This coating exhibits a tensile strength of 20.88 MPa (3.7 times that of pure epoxy), a pencil hardness of 6H, and maintains an impedance modulus exceeding 10^10^ Ω·cm^2^ after prolonged immersion in a 3.5 wt% NaCl solution at pH = 1. It combines outstanding mechanical properties with corrosion resistance. NaCl solution, the impedance modulus remained above 10^10^ Ω·cm^2^, demonstrating both excellent mechanical properties and corrosion resistance.

**Figure 4:**
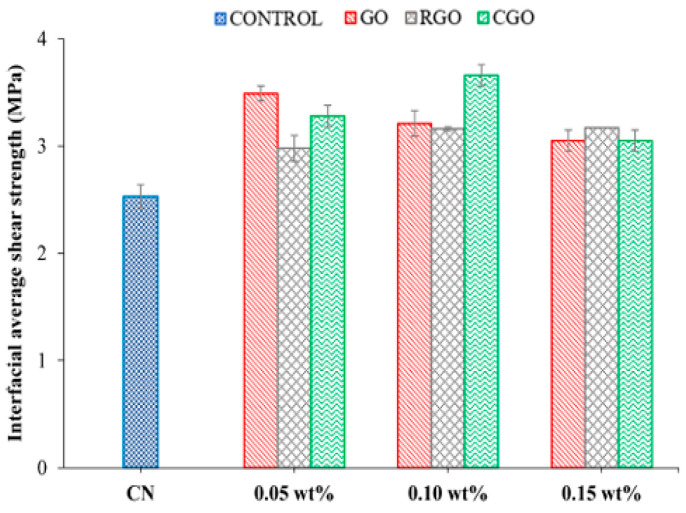
Interfacial average shear strength of various graphene derivative modified composites with different nanofiller concentration [47].

The synergistic effect of cathodic protection and interfacial passivation enhances the protection of metal substrates: When graphene is incorporated into epoxy zinc-rich coatings, conductive graphene forms a continuous conductive network through “electrically conductive bridging” [44]. Upon coating damage, zinc particles act as anodes and corrode preferentially, providing electrochemical protection to the steel substrate (cathode) [45]. Simultaneously, covalent bonding between graphene and epoxy forms a dense interface layer that inhibits the diffusion of corrosion products into the substrate [46], enabling the coating to maintain its anti-corrosion properties even under extreme temperature variations. Kaur et al. [47] investigated the effects of fineness and dosage of steel slag micropowder on the performance of alkyd resin anti-corrosion coatings. As shown in Figure 4, an 800-mesh steel slag powder at 41.5% content yielded optimal comprehensive coating performance: pencil hardness reached 2H, and after 48 h immersion in a 3.5% NaCl solution, the impedance modulus remained at 2.5 × 10^4^ Ω·cm^2^, with maximum protection efficiency of 98.58%. This confirms that steel slag significantly enhances both mechanical and anti-corrosion properties of the coating. Gao et al. [48] developed an organosilicon-modified epoxy non-isocyanate polyurethane (NIPU) anti-corrosion coating using cyclic carbonates and polyoxypropylene diamine (D-230) as raw materials. The optimal formulation achieved a coating solid content of 95%, adhesion grade 0, and hardness 2H, withstanding 168 h of 5% neutral salt spray testing. Combining environmental friendliness, non-toxicity, and outstanding mechanical and anti-corrosion properties, this development offers new insights for green anti-corrosion coating research.

Furthermore, the surface wettability of nanocomposites critically influences their protective performance. Wettability is typically characterized by contact angle (CA), which directly reflects a material’s affinity for liquids such as water or oil. In anti-corrosion coatings, hydrophobic surfaces (CA > 90°) effectively block the penetration of moisture and corrosive media, thereby delaying the corrosion process of the metal substrate [49]. For example, leveraging the hierarchical structure of F-Nb-TiO/SiO nanocomposites, a superhydrophobic surface was achieved with a water contact angle of 155.6° for the composite coating, significantly enhancing both hydrophobicity and corrosion resistance [50]. Wettability depends not only on surface chemical composition but also closely relates to the microscopic roughness introduced by nanofillers. The micro-nano composite structures formed by nanoparticles (e.g., SiO_2_, TiO_2_) on the coating surface can further amplify surface hydrophobic effects, even achieving superhydrophobic states. This is crucial for enhancing coating durability in harsh environments such as humidity and salt spray.

The barrier and synergistic protection mechanisms of the aforementioned two-dimensional nanofillers form the core scientific basis for developing long-lasting anti-corrosion coatings for steel structures (see Section 3.1.1) and high-performance surface protection coatings for concrete (see Section 3.1.2).

#### 2.2.3. Freeze–Thaw Resistance and Thermal Stability

Temperature cycling induces freeze–thaw alternation and thermal shock, which readily leads to stress concentration within materials, triggering microcrack propagation and mechanical property degradation [51]. Nanoparticles suppress thermal stress damage and thermo-oxidative degradation through dual mechanisms of interfacial confinement and structural reinforcement. Their core function lies in enhancing stability by regulating polymer segment motion and interfacial energy transfer.

The enhancement in freeze–thaw resistance stems from microcrack suppression and interfacial toughness improvement: During freeze–thaw cycles, stresses generated by the expansion of ice within internal pore water readily induce crack initiation [52]. Nanoparticles can suppress this process through two pathways: First, nanoparticles fill the pores of the matrix, reducing the space available for ice expansion [53]. Zhang et al. [54] prepared a fluorine-based self-healing superhydrophobic coating through the synergistic interaction between donor-acceptor (D-A) self-assembled polyurethane and hydrophobic metal–organic framework (MOF) nanoparticles. This coating repairs damage within one minute at 80 °C and withstands high-speed water jet impacts of 35 m/s. The SLIPS coating exhibits low ice adhesion strength as low as 11.6 kPa, maintaining stability through multiple cycles while delivering both excellent stability and anti-icing performance. Zhou et al. [55] investigated the influence of nano-silica (NS) and carbon nanotubes (CNTs) on the micro-mechanical properties of the interface transition zone (ITZ) in concrete under freeze–thaw cycles using electron microscopy (EM) and digital image correlation (DIC) techniques. The results indicate that CNTs suppress crack propagation by forming three-dimensional fiber networks, outperforming the pozzolanic reaction and filling effect of NS. This provides theoretical support for enhancing concrete durability in cold regions. Additionally, PEG-coated nanoparticles improve interfacial compatibility, maintain dispersion stability during freeze–thaw cycles, and prevent stress concentration caused by agglomeration.

**Figure 5:**
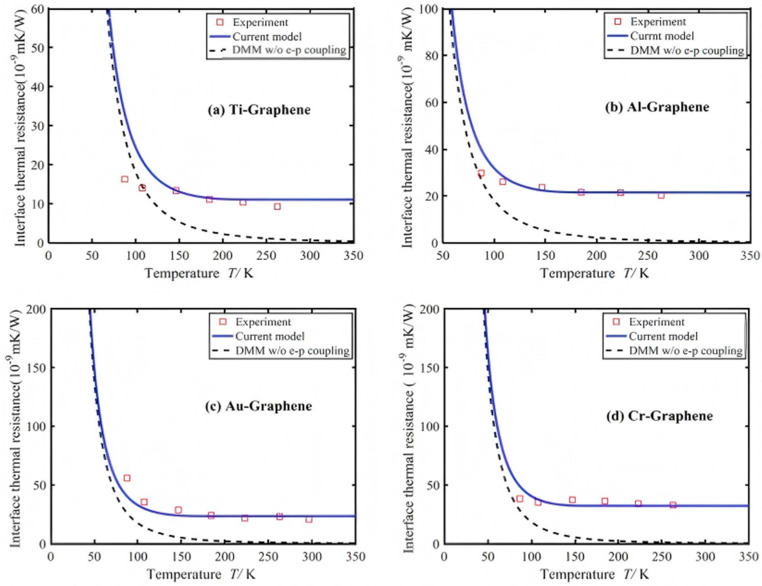
The relationship between the interface thermal resistance of nanocomposite materials and temperature changes [58].

Thermal stability optimization relies on restricting polymer chain motion and enhancing thermal conduction: Strong interface interactions between nanoparticles and the matrix (such as hydrogen bonds and covalent bonds) can form physical crosslinking points, limiting molecular chain thermal motion and elevating both the glass transition temperature (Tg) and thermal decomposition temperature [56]. Kubiak et al. [57] prepared poly(hexamethylene methacrylate) (PHMA)-grafted aluminum oxide nanoparticles (Al_2_O_3_-PGNPs) via surface-initiated atom transfer radical polymerization (SI-ATRP), which were then thermally aged and crosslinked to form highly filled nanocomposites. This material achieves 50 vol% inorganic content, with thermal conductivity over three times higher than pure polymer. It exhibits a maximum modulus of 9.4 GPa and tensile strength of 61.0 MPa, combining processability with outstanding thermal and mechanical properties. Concurrently, conductive fillers like graphene accelerate heat diffusion via phonon conduction, preventing thermal degradation caused by localized overheating. Wang et al. [58] developed an effective medium approximation theory incorporating temperature-dependent interfacial thermal resistance to investigate the effects of temperature and nanofiller orientation on the thermal conductivity of metal/non-metal-based graphene nanocomposites. As shown in Figure 5, graphene nanofillers enhance thermal conductivity by reducing interfacial thermal resistance at elevated temperatures. However, perfect graphene orientation only improves thermal conductivity in the direction parallel to the metal matrix. These findings provide theoretical support for regulating the thermal properties of composites.

It is noteworthy that the porosity of materials directly impacts their freeze–thaw resistance and thermal stability. During freeze–thaw cycles, the expansion of frozen water within pores generates internal stresses, making high-porosity materials more susceptible to microcrack propagation and interfacial debonding. Research indicates that freeze–thaw cycles increase the porosity and degrade the mechanical properties of both ordinary concrete and rubber-modified concrete. However, incorporating rubber and modifying it (e.g., reducing particle size) can suppress porosity growth, enhance freeze–thaw resistance, and improve energy dissipation. Porosity is a key factor in regulating concrete’s freeze–thaw resistance [59]. High porosity significantly reduces thermal conductivity, as air’s thermal conductivity is substantially lower than that of solid matrices and nanofillers. Therefore, nanomodified designs must optimize fillers and process control to minimize detrimental voids, thereby enhancing environmental durability and thermal management capabilities.

As demonstrated by the aforementioned mechanisms, nanofillers of different dimensions exhibit distinct modes of action in enhancing the weather resistance of composite materials: 0D fillers (such as nano-SiO_2_, TiO_2_, and ZnO) primarily improve UV aging resistance through UV absorption, scattering, and catalytic degradation mechanisms, with their performance closely tied to particle size, dispersion, and surface modification. Typical modifications can increase the coating’s UV shielding efficiency by 30–50% and raise its glass transition temperature (Tg) by 5–15 °C. 1D fillers (e.g., carbon nanotubes) leverage their high aspect ratio and conductive networks to primarily enhance the coating’s physical barrier and electrochemical protection functions, demonstrating exceptional performance in corrosion protection and increasing the coating’s impedance modulus by 1–3 orders of magnitude. 2D fillers (e.g., graphene, nanoclay) form labyrinthine barriers through their layered structures, significantly lengthening corrosion medium penetration paths. Simultaneously, synergistic interfacial passivation and hydrophobic modification enhance water resistance and corrosion resistance. Typical additions of 0.1–1.5 wt% can improve salt spray resistance by 2–3 times. In summary, filler dimensions are closely linked to their primary protective mechanisms. Practical applications require targeted selection and composite design based on specific environmental erosion types (UV radiation, moisture, corrosive ions, etc.).

### 2.3. Mechanism of Toughness and Mechanical Property Optimization

The remarkable leap in mechanical properties of nanopolymer composites stems from the synergistic interaction mechanism between nanofillers and polymer matrices. By precisely regulating interfacial interactions and microstructural evolution, these composites achieve coordinated enhancements in toughness, strength, stiffness, and fatigue/impact resistance, providing core support for civil engineering materials to withstand extreme loads and environmental coupling effects. This section systematically explores how nanofillers enhance the mechanical properties of composites under static, dynamic, and cyclic loading through interfacial design, microstructural control, and energy dissipation mechanisms. These properties include toughness, strength, stiffness, fatigue resistance, and impact resistance. Although some mechanisms (e.g., crack inhibition) also manifest in environmental corrosion, this section focuses on analysis from the perspective of mechanical response and fracture behavior (e.g., crack deflection, bridging, matrix shear yielding), complementing the environmental durability enhancement mechanisms discussed in Section 2.2. The following section systematically elucidates the underlying optimization mechanisms across three dimensions: toughening, strength-toughness enhancement, and resistance to dynamic loading.

#### 2.3.1. Toughening Mechanism

The toughening effect of nanocomposites results from the synergistic action of multiple microscopic mechanisms, with the core principle being the regulation of crack propagation pathways and energy dissipation modes through interface design. Crack deflection originates from the modulus disparity between nanofillers and the matrix: when the crack tip encounters high-modulus nanoparticles (such as nano-SiO_2_ or graphene sheets), the stress field becomes distorted, forcing the crack to deviate from its original propagation direction (e.g., along the filler/matrix interface or bending around the filler). This significantly increases both crack propagation length and energy dissipation [25]. Research indicates that in graphene-based composites, layered fillers can increase crack propagation path length by 3 to 5 times and enhance fracture energy by 40% to 80% compared to pure polymers [42].

Crack bridging serves as the core toughening mechanism for fibrous nanofillers (such as carbon nanotubes and nanofibers): When a crack initiates and propagates, nanofibers spanning both sides of the crack bear tensile stress through interfacial adhesion, forming a “bridging effect” that impedes crack opening. When interfacial stress exceeds the adhesion threshold, the nanofibers undergo a “pull-out effect,” overcoming interfacial friction and van der Waals forces in the process, thereby dissipating significant additional energy [20]. Khan et al. [19] demonstrated that when oligomeric imide-modified graphene oxide is composite with epoxy resin, the bridging effect of graphene layers enhances the composite’s fracture toughness (KIC) by 56%. Furthermore, the breaking and reformation of covalent bonds at the interface during pull-out further intensifies energy dissipation.

Crack pinning is primarily dominated by spherical or near-spherical nanoparticles (such as nanoclay or rubber nanoparticles): nanoparticles are fixed within the polymer matrix via physical adsorption or chemical bonding. When a crack propagates to the particle surface, the particle must overcome interfacial bonding forces to detach from the matrix, forming a “pinning point” that impedes crack tip advancement [38]. Furthermore, the size effect of nanoparticles significantly influences pinning efficiency. When particle size matches the dimensions of the plastic zone at the crack tip (typically 50–200 nm), pinning becomes most effective, reducing crack propagation rates by an order of magnitude [56].

Shear yielding of the matrix induced by nanoscale voids represents another critical toughening pathway: When nanoscale fillers with core–shell structures or hollow characteristics (e.g., hollow nano-SiO_2_, polymer-coated nanoparticles) are loaded, plastic deformation or cavitation occurs preferentially in the core-phase regions, forming nanoscale voids. These voids act as stress concentration points, inducing extensive shear yielding in the surrounding matrix and forming broad plastic deformation zones, thereby transforming brittle fracture into ductile fracture [26]. Yuan et al. [38] found that PUCA-grafted SiO_2_ nanoparticles form nanoscale voids in epoxy matrices, increasing the shear-yielding area by threefold and enhancing the composite’s impact toughness by 72%. Uniform void size distribution is crucial for ensuring stable toughening effects.

Additionally, the pore structure of materials exerts a significant dual influence on the mechanical properties of nanocomposites. On one hand, appropriately distributed nanoscale pores or microvoids can serve as stress concentration points, inducing extensive shear yielding in the polymer matrix and thereby enhancing the material’s toughness. On the other hand, excessively high porosity or uneven pore distribution weakens the material’s load-bearing capacity, leading to reduced strength and modulus. Low porosity can enhance tensile/bending strength and thermal conductivity efficiency by densifying the microstructure, whereas high porosity readily induces stress concentration, leading to mechanical property degradation. The uniformity and type (closed/open) of pore distribution further influence performance stability. Optimizing these requires integrating nanofiller dispersion and interfacial interactions, providing critical support for precise material property control [60]. Therefore, in nanocomposite design, precise control over pore structure—including porosity, pore size distribution, and connectivity—must be achieved through optimizing filler dispersion and controlling curing processes. This approach balances strength and toughness to match material requirements.

These multiscale toughening mechanisms are crucial for enhancing the damage tolerance of fiber-reinforced polymer (FRP) composite structural members (see Section 3.3.1) and improving the tensile strain hardening behavior of fiber-reinforced cementitious composites (ECC) (see Section 3.3.2).

#### 2.3.2. Strength and Stiffness Enhancement

The enhancement in strength and stiffness in nanocomposites fundamentally depends on the efficiency of load transfer between nanofillers and the matrix. This efficiency is jointly determined by the quality of interfacial bonding, filler dispersion, and the intrinsic properties of the fillers themselves. Interfacial bonding quality forms the foundation for load transfer: physical adsorption (van der Waals forces, hydrogen bonds) and chemical bonding (covalent bonds, ionic bonds) jointly create a “strong interface,” ensuring efficient load transfer from low-modulus polymer matrices (e.g., epoxy resin modulus~3 GPa) to high-modulus nano-reinforcements (e.g., carbon nanotube modulus ~1 TPa, graphene modulus ~1.05 TPa) [13,61]. Mahendia et al. [18] demonstrated that strong hydrogen bonding between reduced graphene oxide and polyvinyl alcohol enhanced the tensile strength of the composite material by 42% and its elastic modulus by 58%. Furthermore, the efficiency of interfacial stress transfer increased linearly with the density of hydrogen bonds.

The uniform dispersion of nanofillers is essential for effective load transfer: agglomerated nanofillers create stress concentration points, leading to localized overload and premature failure, whereas uniformly dispersed fillers form a three-dimensional reinforcement network that distributes loads evenly throughout the matrix. Liu et al. [42] addressed graphene agglomeration in epoxy resins by modifying graphene with quinacridone via covalent–noncovalent bridging. At a mere 0.2 wt.% loading, the composite achieved a tensile strength of 20.88 MPa—3.7 times that of neat epoxy—demonstrating that optimized dispersion can enhance load transfer efficiency by 2–3-fold.

In practical performance regulation, dispersion uniformity often exerts a greater influence on mechanical properties than filler loading. For example, Liu et al. [42] demonstrated that adding only 0.2 wt% graphene modified with quinone-acridone covalent–noncovalent bridging significantly enhanced the tensile strength of epoxy resin to 3.7 times that of the pure resin (20.88 MPa) due to its excellent dispersion. This reinforcement efficiency was markedly higher than that of systems with high loading but poor dispersion. This demonstrates that optimizing dispersion processes and enhancing interfacial compatibility can achieve more pronounced performance improvements at low loading levels while reducing performance fluctuations and premature failure risks caused by agglomeration.

The geometric morphology and size effects of reinforcing particles significantly influence load transfer efficiency: One-dimensional nanofibers (e.g., carbon nanotubes) exhibit a “fiber reinforcement effect” due to their high aspect ratio (typically >100), where loads are transferred along the fiber axis, and their reinforcement efficiency follows the Rule of Mixtures; Two-dimensional sheet fillers (e.g., graphene, nanoclay) reinforce the matrix through in-plane stress transfer. Their enhancement effect correlates with sheet orientation, with the most pronounced stiffness increase occurring when sheets are parallel to the load direction]. Zero-dimensional nanoparticles (such as nano-SiO_2_) uniformly enhance the overall strength of the matrix through point-like reinforcement [38]. Furthermore, the size effect of nano-reinforcements cannot be overlooked. When particle size is reduced to the nanoscale, quantum confinement effects further enhance the particle’s inherent strength and modulus, providing stronger “support points” for load transfer [33].

#### 2.3.3. Fatigue Resistance and Impact Performance

The optimization of fatigue and impact resistance in nanocomposites fundamentally involves constructing efficient dynamic energy dissipation mechanisms through interfacial regulation and microstructural design to withstand damage from cyclic and transient impact loads. Enhanced fatigue resistance stems from energy dissipation and self-healing under cyclic loading: During fatigue cycles, the interface region between nanofillers and the matrix continuously dissipates cyclic energy through friction slip, hydrogen bond rearrangement, and covalent bond breakage-reformation, thereby delaying microcrack initiation and propagation [13]. Seraji et al. [20] found that epoxy-infiltrated modified carbon nanotube fiber composites exhibited a 63% increase in fatigue strength under 10^6^ cyclic loads compared to the original fibers. Friction slip in the interface region dissipated approximately 40% of the cyclic energy, effectively suppressing fatigue crack propagation. Furthermore, the three-dimensional network formed by nanofillers can impede microcrack connectivity, transforming concentrated damage into dispersed microdamage and extending the material’s fatigue life [55].

Impact resistance optimization relies on rapid energy dissipation under transient impact loads: When subjected to impact, nanocomposites achieve rapid energy conversion and dissipation through multiple pathways. First, tensile fracture and pull-out of fibrous nanofillers (such as carbon nanotubes) consume impact energy. Second, matrix shear yielding and plastic deformation induced by nanoparticles convert impact kinetic energy into plastic deformation energy. Third, dynamic friction and chemical bond breakage at the interface further dissipate energy [25]. Zhang et al. [54] in the prepared fluorine-based self-healing superhydrophobic coating, the synergistic interaction between hydrophobic metal–organic framework nanoparticles and the polyurethane matrix enables the coating to maintain structural integrity under high-speed water jet impact at 35 m/s. Impact energy is efficiently dissipated through three pathways: particle extraction, matrix shear yielding, and interfacial friction.

It is noteworthy that enhancing fatigue resistance and impact performance requires balancing toughness and strength: a single toughening strategy (such as excessive addition of rubber nanoparticles) may lead to strength reduction, whereas the synergistic optimization of “strength-toughness-resistance to dynamic loading” can be achieved through the combination of multiple nanofillers (e.g., carbon nanotubes + nano-SiO_2_) [7]. For example, Chintalapudi et al. [6] incorporated graphene oxide and nano-SiO_2_ into cement-based composites. The resulting material not only exhibited a 35% increase in compressive strength but also retained 82% of its strength after 105 fatigue cycles, with impact toughness improving by 59%. This offers a viable solution for enhancing the resistance of civil engineering structures to dynamic loads.

From the perspective of toughness enhancement and mechanical property optimization mechanisms, nanofillers of different dimensions synergistically improve composite material performance through distinct microstructural mechanisms: 0D fillers (such as nano-SiO_2_ and rubber particles) primarily enhance toughness through crack pinning, inducing matrix shear yielding, and pore regulation. Typical toughening effects can increase impact toughness by 50–100% while simultaneously uniformly enhancing matrix strength as point-like reinforcing phases. 1D fillers (e.g., carbon nanotubes, nanofibers) leverage high aspect ratios to achieve efficient crack bridging and fiber pull-out effects, serving as key enhancers of fracture toughness and fatigue resistance. They typically increase composite fracture toughness (KIC) by 40–80% and extend fatigue life by several orders of magnitude. 2D fillers (e.g., graphene, nanoclay) simultaneously enhance strength and stiffness through layer orientation control, crack deflection, and interfacial strengthening. Typical additions of 0.2–1.0 wt% can increase tensile strength by 200–300% and elastic modulus by 50–150%. Synergistic use of multi-dimensional fillers (e.g., 1D+2D, 0D+2D) enables balanced optimization of strength, toughness, and dynamic load resistance, providing multi-dimensional strategies for performance design of civil engineering materials under extreme conditions. In summary, nanofillers of different dimensions enhance the weather resistance and mechanical properties of composites through unique interfacial interactions. Their core mechanisms, performance contributions, and application orientations are summarized in Table 2.

## 3. Application Advances in Key Civil Engineering Materials

Building upon the core mechanisms elucidated in Chapter 2—where nano-interface modification synergistically enhances material weather resistance and toughness—this chapter will focus on the practical application of these mechanisms in key civil engineering materials. It will reveal the implementation pathway from fundamental principles to material performance and functional realization.

Nanotechnology offers a novel approach to addressing traditional limitations in strength, durability, and functionality of civil engineering materials by designing them at the atomic and molecular scale [62]. As the primary civil engineering material, enhancing the performance of concrete is the core focus of related research [63,64]. By incorporating nanoparticles (such as nano-SiO_2_, carbon nanotubes, etc.), the microstructure of concrete can be effectively optimized: nanoparticles serve both as ultrafine fillers to densify the matrix and as active centers to promote hydration, thereby significantly enhancing the material’s mechanical properties and durability metrics such as water resistance and corrosion resistance [64,65]. More importantly, nanotechnology is driving materials toward high performance and sustainability. By enabling equivalent or superior performance with reduced cement usage, nanomodification aligns with green building principles. Simultaneously, this technology is giving rise to a new generation of materials featuring intelligent properties such as self-cleaning and self-sensing capabilities [66].

**Figure 6:**
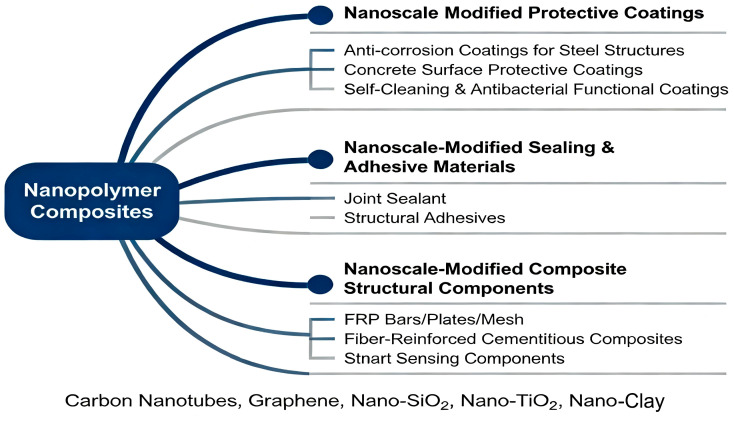
Key applications of nanocomposites in civil engineering.

This chapter reviews the application of nanotechnology in key civil engineering materials. The following sections will focus on three areas that have achieved significant progress: nanomodified protective coatings, nanomodified sealing and bonding materials, and nanomodified composite structural components while analyzing the underlying mechanisms behind their enhanced performance. The schematic diagram is shown in Figure 6.

### 3.1. Nanoscale Modified Protective Coatings

Traditional concrete coatings generally include organic coatings, inorganic coatings, and organic–inorganic composite coatings. Although coating methods are simple, easy to implement, and effective, they present various issues. For example: traditional organic coatings offer good integrity, high density, and superior protective performance, but generally suffer from susceptibility to aging and poor environmental compatibility; traditional inorganic coatings typically exhibit good aging resistance, but are prone to cracking and provide inferior protective performance [67]. As the service environments of civil engineering structures grow increasingly complex, traditional protective coatings are gradually failing to meet the demands for long-term protection and functionalization. The introduction of nanotechnology offers novel modification pathways for protective coatings. Incorporating nanomaterials into coatings significantly enhances their density, impermeability, hydrophobicity, water resistance, and UV resistance. A wide range of nanomodified coatings have already found extensive application in the durable protection of metal structures [68].

#### 3.1.1. Anti-Corrosion Coatings for Steel Structures

Steel structures are highly susceptible to rusting in corrosive environments such as humidity and salt spray, severely compromising their load-bearing capacity and service life. Nanomodified anti-corrosion coatings effectively delay the erosion of the substrate by corrosive media by enhancing the coating’s barrier properties, adhesion, and inherent stability. The epoxy/graphene coating system incorporates functionalized graphene (typically 0.5–1.5 wt.%) to leverage its exceptional barrier properties stemming from its two-dimensional layered structure (see Section 2.2.2). This creates a labyrinthine barrier within the coating, thereby extending its salt spray protection time by 2–3 times [69]. Simultaneously, graphene’s interfacial reinforcement effect (see Section 2.3.2) also contributes to enhancing the coating’s mechanical strength and adhesion.

At the engineering application level, Qin et al. [69] compared the corrosion resistance enhancement effects of two-dimensional graphene sheets (G) and three-dimensional porous graphene (3DG) on zinc-rich epoxy (ZRE) coatings. The results indicate that the optimal G content for modifying ZRE coatings is 0.5 wt%. However, leveraging its interconnected porous structure and high crystallinity, 3DG significantly optimizes coating performance at just 0.1 wt%: its corrosion current density dropped to 1.9 × 10^−7^ A·cm^−2^, representing only one-tenth of the G/ZRE coating with equivalent G content and 27% of the optimal G/ZRE coating. Charge transfer resistance reached 5.99 × 10^5^ Ω·cm^−2^ (46 times that of the optimal G/ZRE coating). A 300 h immersion test confirms superior long-term stability of the 3DG/ZRE coating, with no formation of the detrimental corrosion product Zn_5_(CO_3_)_2_(OH)_6_. 3DG enhances corrosion resistance by promoting compatibility with epoxy resin, constructing a zinc flake conductive network, and strengthening the physical barrier effect. This approach offers new insights for developing high-performance corrosion-resistant coatings with low filler loading and can be extended to other two-dimensional filler modification systems.

In the field of oil and gas extraction, Chen et al. [70] designed a pH-responsive smart epoxy coating with both active and passive corrosion protection capabilities. This coating encapsulates the corrosion inhibitor 2-mercaptobenzothiazole (MBT) using ZIF-67 (zeolite imidazole framework material), and is modified by combining sepiolite (SEP) with perfluorooctyl triethoxysilane (PFTS) to prepare SEP-modified MBT. 2-Mercaptobenzothiazole (MBT)-loaded zeolite imidazole framework (ZIF-67) was combined with sepiolite (SEP), and the obtained hybrid was further modified with perfluorooctyl triethoxysilane (PFTS) for the preparation of the SEP-ZIF@MBT composite filler. This filler was then used to construct the SEP-ZIF@MBT/EP composite coating. The characterization results indicate that ZIF-67 exhibits a loading efficiency of 17.38% for MBT and demonstrates sensitive responsiveness to pH changes, achieving a cumulative MBT release rate of 89.74% in acidic environments. PFTS modification increased the coating contact angle from 68.03° to 118.13°, imparting excellent hydrophobicity. Simultaneously, coating adhesion improved by 91.39% compared to pure epoxy, reaching 10.45 MPa. Electrochemical impedance spectroscopy (EIS) testing indicated that in acidic corrosive environments, composite coatings containing approximately 0.5 wt% of intelligent corrosion inhibitor fillers demonstrate long-term stable protective performance. Their low-frequency impedance remains at a high level throughout immersion tests lasting several dozen days (typically 2–3 orders of magnitude superior to pure epoxy coatings), indicating sustained corrosion inhibition capability. The corrosion protection mechanism stems from synergistic multi-effect interactions: SEP’s layered chain structure and high water absorption form a physical barrier; ZIF-67 actively releases MBT in response to pH changes induced by corrosion; Co^2+^ competes with Fe^2+^/Fe^3+^ for OH^-^ to form a protective film; PFTS modification enhances coating hydrophobicity and suppresses ZIF-67 structural collapse. This study provides innovative insights and experimental support for developing highly efficient, durable smart anti-corrosion coatings.

#### 3.1.2. Concrete Surface Protective Coatings

**Figure 7:**
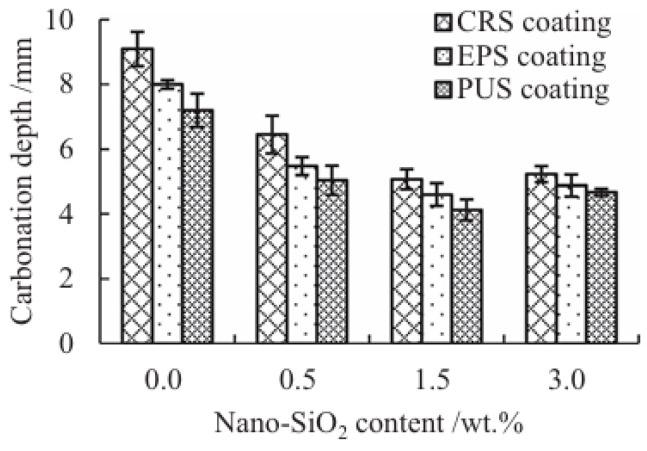
Carbonation depths of concrete with nano-SiO_2_ modified coatings [72].

Composite anti-carbonation materials represent a new trend in protecting reinforced concrete structures, with their research and application gaining increasing attention. This approach employs composite technology to combine two or more distinct materials, enhancing their anti-carbonation properties. By integrating the advantages of inorganic and organic anti-carbonation materials, it forms a multi-layered protective system that effectively blocks carbon dioxide penetration while providing excellent crack resistance and durability. For instance, incorporating fiber materials into concrete improves the carbonation resistance, crack resistance, permeability resistance, and freeze–thaw resistance of reinforced concrete structures while broadening the material’s application scope. Furthermore, nanocomposites demonstrate significant potential in carbonation resistance. Incorporating nanoparticles further enhances material durability and environmental adaptability. Experiments indicate that adding 1% carbon nanotubes to epoxy resin coatings can improve carbonation resistance by over 30%. In practical engineering applications, composite anti-carbonation materials require specialized preparation processes and construction techniques to ensure optimal performance. During construction, environmental conditions such as temperature and humidity must be strictly controlled to guarantee coating uniformity and adhesion. Li et al. [71] investigated the effect of nano-SiO_2_ on the long-term carbonation resistance of concrete coated with three polymers: polyurethane, epoxy resin, and chlorinated rubber. Research indicates that the optimal loading of nano-SiO_2_ typically ranges between 1 wt% and 2 wt%. This concentration effectively fills micro-defects in coatings, enhances density, mitigates UV aging damage, and achieves a carbonization resistance efficiency of 43–44%. Based on long-term performance prediction models, an appropriate amount of nano-SiO_2_ can extend the service life of polymer coatings by approximately 50% to 80%, with specific effects depending on the substrate type and environmental conditions, as shown in Figure 7 below. Chen et al. [72] investigated the coupled effects of temperature, moisture content, and nano-SiO_2_ content on the rutting resistance of AC-13 graded nano-SiO_2_-modified asphalt concrete. The results indicate that temperature and moisture content are the primary influencing factors. Increases in both significantly reduce dynamic stability, with dynamic stability decreasing approximately linearly with moisture content at the same temperature. Nano-SiO_2_ content has a negligible effect, with an optimal dosage of approximately 0.4%. This study provides reference for asphalt pavement material design in hot and humid environments.

**Figure 8:**
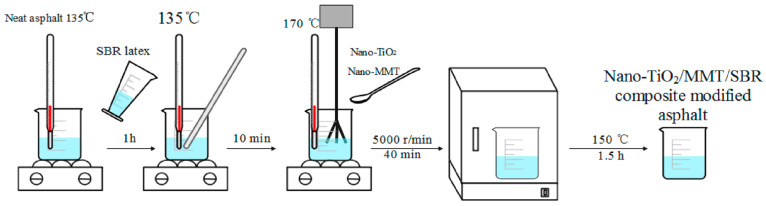
Flow chart of modified asphalt preparation [73].

Additionally, both modified and unmodified nano-TiO_2_ can effectively enhance the carbonation resistance of coated concrete, with modified nano-TiO_2_ demonstrating superior improvement effects. Liao et al. [73] conducted research on nano-TiO_2_ coating materials. To enhance the high-temperature resistance and UV aging resistance of SBR-modified asphalt in the harsh, high-altitude, and intense UV environment of western China, this study employed BBD optimization to formulate nano-TiO_2_/MMT/SBR composite modified asphalt via high-shear mixing. The optimal dosage was determined as 1.5% nano-TiO_2_, 4% MMT, and 6% SBR latex (60% solid content). As shown in Figure 8, the synthesis process was analyzed via DSR, BBR, SEM, and FTIR. Nano-TiO_2_ absorbs and reflects UV radiation, while MMT forms an oxygen-blocking network. Their synergistic action delays the conversion of light components into asphaltic material, stabilizing the CMI value around 2. The stiffness modulus change rate decreased to 0.13, and the carbonyl index decreased from 5.16 to 0.28. This provides support for selecting asphalt pavement materials in cold regions with intense ultraviolet radiation.

#### 3.1.3. Self-Cleaning and Antibacterial Functional Coatings

**Figure 9:**
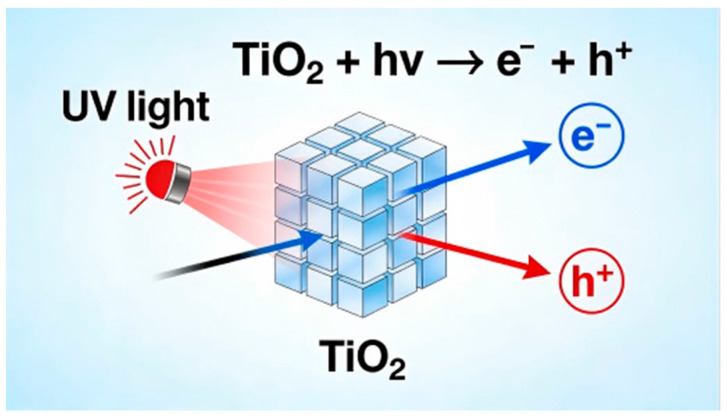
Photocatalytic activity of titanium dioxide coatings.

To enhance maintenance efficiency and hygienic safety in civil engineering structures such as building facades, bridges, and tunnels, nanocoatings with self-cleaning or antibacterial properties have garnered significant attention. Self-cleaning coatings primarily rely on two mechanisms: photocatalytic (e.g., nano-TiO_2_) and superhydrophobic types. Nano-TiO_2_ generates highly oxidative free radicals under UV excitation, decomposing surface organic contaminants; simultaneously, its hydrophilicity enables uniform water film spreading, facilitating rainwater removal of stains. Superhydrophobic coatings achieve contact angles exceeding 150° by constructing nano-micron scale rough structures and modifying low-surface-energy substances (e.g., fluorinated silanes), causing water droplets to roll off while carrying away dust. Combining both mechanisms yields synergistic effects. Due to its outstanding photocatalytic properties, nano-titanium dioxide (TiO_2_) is extensively applied in surface treatments for architectural decorative materials. Under UV irradiation, TiO_2_ generates electron-hole pairs where holes (h^+^) oxidize organic pollutants, while electrons (e^−^) react with oxygen to produce reactive species like ·OH, enabling photodegradation of contaminants. As shown in Figure 9, rough surfaces effectively enhance the affinity between photocatalytic coatings and water molecules, improving surface wettability and self-cleaning efficiency. Furthermore, TiO_2_–WO_3_ heterojunction composites forming nanoscale films enhance carrier separation efficiency. Under UV irradiation, these films exhibit outstanding hydrophilicity and catalytic activity, making them suitable for glass curtain walls and exterior building facades.

Micro-nano structures can effectively mimic the superhydrophobic properties of natural surfaces like lotus leaves. By employing techniques such as nanoimprinting and laser etching, they create synergistic structures with dual roughness at the micrometer and nanometer scales on architectural decorative materials. This results in contact angles exceeding 160° and rolling angles below 5°, achieving a self-cleaning effect for water droplets [74]. This type of surface significantly reduces dust and contaminant adhesion, enhancing the self-cleaning properties and durability of building facades. It is particularly suitable for high-end decorative materials such as curtain wall glass and ceramic cladding.

Wettability exhibits a close synergistic relationship with surface roughness, typically described by the Wenzel or Cassie-Baxter models. In nanocomposite coatings, the introduction of nanofillers such as TiO_2_ and SiO_2_ not only alters surface chemistry but also significantly influences the coating’s apparent contact angle and rolling angle by constructing multiscale rough structures [75]. For instance, SiO_2_/TiO_2_ heterojunction composite coatings exhibit superhydrophilicity under UV irradiation, attributed to the high specific surface area and surface hydroxylation formed by nanoparticles [76]. This tunable wettability provides a theoretical basis for developing multifunctional coatings combining self-cleaning, anti-icing, and anti-fouling properties. In practical engineering applications, coating wetting behavior and surface morphology must be optimized according to service environments (e.g., rainwater runoff, oil contamination, icing conditions) to achieve long-term functionality.

Nano-silver (Ag) and nano-zinc (Zn) demonstrate outstanding performance as highly effective broad-spectrum antimicrobial agents in architectural decorative coatings. They release Ag^+^ and Zn^2+^ ions that bind to bacterial cell membrane proteins and DNA, disrupting metabolic processes and compromising cell integrity to achieve efficient elimination of bacteria and fungi [77]. When the particle size of silver nanoparticles is controlled within the range of 10 nm to 20 nm, their specific surface area significantly increases. This enables rapid penetration of cell membranes, inducing reactive oxygen species (ROS) production and triggering cell apoptosis. Consequently, it effectively inhibits the proliferation of surface microorganisms, mold, and algae, making it suitable for hygiene-sensitive environments such as hospital buildings and underground structures. Current challenges lie in maintaining long-term antimicrobial activity and achieving stable immobilization of the nanoparticles. Depending on the specific protective objectives, nanofillers play differentiated roles within coatings, achieving significant performance enhancements through optimized addition levels. Table 3 summarizes the typical parameters and effects of major nanomodified protective coating systems.

### 3.2. Nanoscale-Modified Sealing and Adhesive Materials

Sealing and bonding materials are critical auxiliary materials that ensure the integrity, watertightness, and durability of engineering structures. Nanotechnology modification can significantly enhance their overall performance.

#### 3.2.1. Joint Sealant

**Figure 10:**
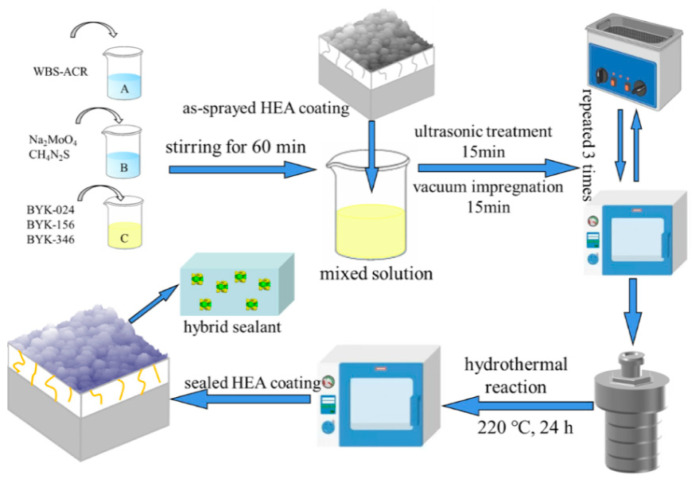
The schematic diagram of the preparation of MoS_2_/WBS-ACR@HEA coating [79].

Seam sealants must endure prolonged exposure to temperature cycling, load deformation, and environmental corrosion. Traditional materials are prone to aging, hardening, or adhesive failure. Nanoparticles, acting as reinforcing phases, enhance the sealant’s tensile strength, tear resistance, and elastic recovery rate. Mengistu et al. [78] combined α-Fe_2_O_3_ nanorods synthesized via coprecipitation with laser-induced graphene (LIG) electrodes to construct a chemical resistive sensor for detecting 1-butanol at room temperature. At 55% relative humidity, the sensor exhibited a response of 185 ± 25% to 100 ppm 1-butanol, with a detection limit of 36 ± 11 ppm. It demonstrated rapid response/recovery and could distinguish different VOCs based on response characteristics, combining low energy consumption with high selectivity. Shi et al. [79] developed a one-step hydrothermal method to in situ prepare MoS_2_/water-based silicone-modified acrylic resin (WBS-ACR) hybrid sealants within defects of plasma-sprayed high-entropy alloy (HEA) coatings, as shown in Figure 10 below. This sealant exhibits a penetration depth exceeding 180 μm, combining excellent wear resistance (with a wear rate as low as 9.2 × 10^−5^ mm^3^/Nm) and corrosion resistance (with a corrosion current two orders of magnitude lower than pure MoS_2_ sealants), offering a novel solution for thermal spray coating applications in extreme environments.

Wang et al. [80] synthesized water-based silicone-modified acrylic sealant (WAS) via emulsion polymerization and employed vacuum ultrasonic immersion to seal plasma-sprayed Fe-based amorphous coatings. The sealant achieved a penetration depth exceeding 70 μm with excellent thermal stability (maximum decomposition temperature of 409 °C). After 34 days of salt immersion, the coating’s corrosion current density decreased by more than one order of magnitude while maintaining over 90% protection efficiency, offering a novel approach for environmentally friendly coating sealing.

#### 3.2.2. Structural Adhesives

The incorporation of nanomaterials can effectively enhance the mechanical properties and heat resistance of epoxy adhesives. Nanoparticles such as nano-silica and nano-alumina can significantly improve the tensile strength and toughness of epoxy resins [81]. Compared to traditional materials such as metals or polymers, nanomaterials offer numerous advantages. They are lightweight, providing enhanced mechanical properties, higher specific strength, and durability. They exhibit high thermal stability, low thermal expansion coefficients, and efficient heat dissipation capabilities. Additionally, they can be tailored with specific chemical properties to achieve enhanced performance, such as resistance to environmental degradation, while offering multifunctionality in applications with fewer components, manufacturing techniques, and design flexibility. These characteristics make composite materials highly valuable for applications across numerous industries [82]. Currently, researchers are working to develop novel nanomodified epoxy resin adhesives to further enhance their performance [83].

**Figure 11:**
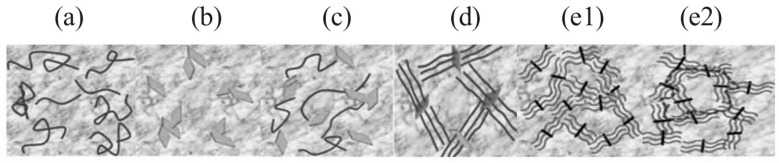
Schematic diagram of reinforcement dispersion in epoxy composite materials (**a**) Carbon nanotubes; (**b**) gold nanoparticles (GNP); (**c**) CNT+GNP composite; (**d**) CNT-GNP hybrid; (**e1**,**e2**) evolution of the conductive network during tensile loading [84].

Li et al. [84] modified epoxy resin by dispersing a hybrid filler composed of carbon nanotubes (CNTs) grown on graphene nanosheets (GNPs) into the epoxy matrix. The NT-GNP/epoxy composite exhibited unique self-sensing behavior, enabling effective in situ monitoring of irreversible permanent deformation. As shown in Figure 11, embedding the CNT-GNP hybrids into unmodified epoxy resin achieves optimal dispersion of CNTs and GNPs, along with enhanced interfacial adhesion between the carbon filler and matrix, thereby significantly improving load transfer efficiency. Uniformly dispersed CNTs on GNPs can form a homogeneous CNT network while also suppressing GNP stacking and aggregation.

### 3.3. Nanoscale-Modified Composite Structural Components

#### 3.3.1. FRP Bars/Plates/Mesh

The performance of FRP products largely depends on the interfacial bonding between fibers and the resin matrix. Nanofillers act as “bridges”, enhancing both mechanical interlocking and chemical bonding at the interface. Carbon nanotubes (CNTs), with their high aspect ratio and outstanding mechanical properties, have become a research hotspot. Grafting CNTs onto carbon fiber surfaces or dispersing them within the resin significantly increases both interfacial shear strength (IFSS) and interlaminar shear strength (ILSS). CNTs increase surface roughness and reactivity of fibers, while their inherent network structure effectively transfers stress, inhibiting microcrack initiation and propagation. Additionally, CNTs improve FRP’s electrical conductivity, thermal conductivity, and damping properties, endowing it with smart potential such as structural health monitoring.

To further enhance the interlaminar properties of composite materials, researchers have recently begun exploring a novel approach: pre-assembling CNTs into macroscopic films and introducing them into the composite interlaminar space via intercalation. Zeng et al. [85] investigated the influence of gelatin-modified carbon nanotubes (g-CNTs) on the mechanical properties of carbon fiber-reinforced polymer (CFRP) composites through both fiber-interface enhancement and matrix enhancement mechanisms. For fiber reinforcement, g-CNTs were uniformly coated onto carbon fiber surfaces via electrophoretic deposition (EPD) at 0.1 mg/mL concentration and 10 V/cm voltage for 2 min. This increased the interfacial normal strength (IFNS) by 40.3%, while CFRP flexural strength and modulus improved by 21.9% and 25.3%, respectively. In matrix reinforcement, 0.1 wt% g-CNTs uniformly dispersed in epoxy resin increased IFNS by 12.6%, with flexural strength and modulus improving by 20.3% and 11.4%, respectively. Both approaches enhance performance by improving CNT dispersion and strengthening fiber–matrix interface bonding (mechanical interlocking and chemical interaction). The g-CNTs demonstrated superior effectiveness compared to carboxylated CNTs, offering an eco-friendly and efficient solution for multifunctional reinforcement in advanced CFRP composites.

#### 3.3.2. Fiber-Reinforced Cementitious Composites

Engineered Cementitious Composites (ECCs) are renowned for their high ductility and multi-crack propagation characteristics, with their toughness primarily stemming from the bridging effect of polymer fibers. Introducing nanoscale polymer fibers (such as nano-nylon and polyvinyl alcohol fibers) alongside conventional micron-scale fibers creates a multiscale reinforcement system, enabling synergistic toughening at both microscopic and macroscopic levels. Nanofibers effectively bridge microcracks, delaying crack initiation and early propagation, while micron-scale fibers control macro crack width. This synergistic interaction endows the composite with more stable strain-hardening behavior and higher ultimate tensile strain.

In enhancing the toughness of cementitious materials, the combined use of fibers and nanomaterials demonstrates significant advantages. Currently, steel fibers and polyvinyl alcohol (PVA) fibers are commonly used reinforcing materials: steel fibers, with their high elastic modulus and tensile strength, can significantly improve material strength and toughness, but suffer from high density and cost; PVA fibers, characterized by low weight and cost, effectively suppress early plastic shrinkage cracks but offer limited strength enhancement. Research indicates that co-incorporating nano-SiO_2_ with steel–PVA hybrid fibers into epoxy-based cementitious repair materials achieves synergistic reinforcement effects [86,87]. On one hand, nano-SiO_2_ promotes cement hydration, increases the formation of calcium silicate hydrate gel, fills matrix pores, and enhances density. On the other hand, fiber incorporation suppresses crack initiation and propagation, improving the integrity of the matrix. Hu et al. [88] discovered through computed tomography experiments that nano-SiO_2_ with appropriate content, when combined with fibers, can optimize the internal pore structure of the material and enhance the interfacial adhesion between the fibers and the matrix. Fan et al. [89] investigated the effect of nano-SiO_2_ on the bond strength between recycled aggregate concrete (RAC) and reinforcing steel through pull-out tests, microscopic analysis, and acoustic emission monitoring. The results indicate that nano-SiO_2_ effectively fills internal pores and cracks within RAC. At a 3% dosage, porosity decreased by 36.96%, significantly enhancing bond strength. The established multi-factor coupled bond strength calculation formula and constitutive model showed good agreement with the experimental and numerical simulation results, providing support for RAC structural design.

Furthermore, the incorporation of nanofibers has minimal impact on the workability of the matrix while optimizing the fiber–matrix interface transition zone and reducing defects. Future research should focus on the dispersion process of nanofibers and their performance evolution under long-term loading and environmental effects to advance the practical application of such materials in seismic resistance, blast resistance, and restoration engineering. To visually demonstrate the substantial improvement in mechanical properties of key civil engineering materials achieved through nanoscale modification, Table 4 presents representative quantitative examples of performance leaps realized via interfacial engineering—from polymer-based to cement-based systems.

## 4. Challenges and Future Outlook

Although nanopolymer composites have demonstrated tremendous potential in civil engineering applications, a series of critical challenges remain on their path from laboratory development to large-scale engineering implementation. These challenges encompass not only technical difficulties inherent to material science itself, but also encompass multiple dimensions such as refining performance evaluation systems, demonstrating economic viability, and addressing environmental safety considerations. Overcoming these obstacles and clarifying future development directions are prerequisites for achieving breakthrough applications of this technology and driving innovation in civil engineering materials. Currently, research on nanocomposites is at a pivotal stage of transitioning from fundamental studies to engineering applications. It urgently requires collaborative efforts among academia, industry, and engineering communities to jointly overcome technical bottlenecks, establish comprehensive evaluation systems, advance standardization processes, and ultimately achieve large-scale deployment in infrastructure construction and maintenance. This will provide material support for building a safer, more durable, and smarter modern civil engineering system. Although nanomodification demonstrates tremendous potential, different material systems exhibit distinct advantages, disadvantages, and applicable scenarios when advancing toward engineering applications, each facing unique challenges. Table 5 provides a comprehensive assessment of these aspects, offering context for the subsequent in-depth analysis of specific challenges.

### 4.1. Current Key Challenges

#### 4.1.1. Technical Bottlenecks

The uniform dispersion of nanofillers represents the primary technical challenge in the preparation of nanocomposites. Nanoparticles such as carbon nanotubes, graphene, and nano-silica, due to their extremely high specific surface area and surface energy, readily agglomerate, forming difficult-to-separate “tangles” or “agglomerates” [90]. This agglomeration phenomenon primarily stems from the combined effects of van der Waals forces, electrostatic interactions, and surface tension, making it difficult to achieve an ideal monodisperse state for nanoparticles within the polymer matrix. Such non-uniform dispersion not only fails to leverage the unique interfacial effects of nanomaterials—such as reinforcement and toughening—but may also create defects within the matrix. These defects act as stress concentration points, triggering the initiation and propagation of microcracks during service, leading to a decline or even degradation in material performance. More critically, agglomerates create weak interface zones within the material, significantly diminishing the composite’s mechanical properties, weather resistance, and durability. This compromises its long-term service reliability in civil engineering applications [91].

To overcome this challenge, researchers have developed various physical and chemical dispersion methods. Physical methods primarily include mechanical approaches such as ultrasonic dispersion, high-shear mixing, and ball milling, which apply external energy to break apart agglomerates of nanoparticles. Among these, ultrasonic dispersion is the most commonly used technique. It effectively disperses nanofillers through the cavitation effect and microjet action generated by ultrasonic waves. However, it has limitations including high energy consumption, lengthy processing times, and the potential to damage the structure of nanomaterials [92]. High-shear mixing is suitable for industrial production, but process parameters such as shear rate, processing time, and temperature must be optimized to ensure dispersion effectiveness while preventing material degradation. Chemical methods focus on enhancing the dispersion stability of nanofillers through surface modification. Common strategies include functionalizing nanoparticle surfaces with coupling agents to introduce polymer–matrix-compatible functional groups, thereby strengthening interfacial interactions; or introducing polymer chains onto nanoparticle surfaces via surface graft polymerization to form “brush-like” structures that prevent agglomeration through steric hindrance effects [93]. Additionally, in situ polymerization can be employed to polymerize monomers in the presence of nanoparticles, enabling the nanoparticles to be encapsulated or grafted during the polymerization process, thereby achieving superior dispersion.

The uneven dispersion of nanofillers not only poses a challenge in the preparation process but is also the primary cause of significant performance fluctuations and poor reproducibility in composite materials. Agglomerates form weak interface zones and micro-defects within the matrix, accelerating performance degradation during service and complicating lifespan prediction. In contrast, even filler systems with slightly inferior properties but excellent dispersion often exhibit superior overall durability and reliability compared to high-performance fillers whose poor dispersion creates “performance bottlenecks.” Therefore, establishing performance prediction models based on dispersion quality and promoting standardized processes are crucial steps toward advancing nanocomposites into engineering applications.

However, despite some progress achieved at the laboratory scale, achieving long-term, stable, and uniform dispersion of nanofillers within the polymer matrix remains the primary hurdle in preparing high-performance composites [94]. First, the surface modification and dispersion processes for nanofillers are complex and costly, making it difficult to meet the economic requirements of industrial-scale production. Second, dispersed nanofillers tend to re-agglomerate during storage, processing, and use, making the maintenance of long-term dispersion stability a critical issue. Third, the compatibility between different nanofillers and polymer matrices varies significantly, necessitating the development of customized dispersion strategies for specific systems. Fourth, laboratory-scale dispersion techniques face challenges in direct scaling to industrial continuous production, necessitating solutions for equipment selection, process parameter optimization, quality control, and other engineering issues. Therefore, developing efficient, low-cost, and industrializable dispersion technologies for nanofillers, alongside establishing corresponding evaluation standards and specifications, is crucial for advancing the large-scale application of nanopolymer composites in civil engineering.

Long-term interface stability is one of the core technical challenges facing nanocomposites in civil engineering applications [95]. The interface region between nanofillers and the polymer matrix is critical for load transfer, stress distribution, and functional realization. Its performance directly determines the overall mechanical properties, durability, and service life of the composite material. Under prolonged exposure to complex civil engineering environments, this vital interface faces severe challenges.

Additionally, controlling porosity represents another critical process challenge in the large-scale production of nanocomposites. During processes such as melt blending and curing molding, agglomeration of nanofillers, solvent evaporation, or reaction byproducts may introduce microvoids. These voids not only serve as stress concentration points that compromise mechanical property uniformity but also provide diffusion pathways for moisture and corrosive media, accelerating material aging. Future process development should focus on: ① developing low-porosity in situ polymerization or vacuum-assisted molding processes [96]; ② utilizing the barrier effects of nanofillers (e.g., nanoclay, graphene) to suppress pore formation [97]; and ③ establishing quantitative predictive models linking pore structure to performance to guide process optimization [98]. Through multiscale pore regulation, the long-term service reliability of nanocomposites under extreme conditions can be further enhanced.

Wettability is also a key factor affecting interfacial adhesion and long-term durability of nanocomposites. In coatings, sealants, or structural adhesives, the wettability of substrate surfaces directly influences the dispersion state of nanofillers and interfacial bonding strength. If substrate surface energy is too low or wettability is poor, nanofillers tend to agglomerate, leading to interfacial defects and stress concentration. Furthermore, in humid or underwater environments, the contact angle and penetration rate of water on material surfaces determine medium ingress behavior, subsequently affecting the chemical stability and mechanical properties of the interfacial layer. Future research should systematically evaluate the dynamic wetting behavior of nanocomposites under varying environmental conditions (humidity, temperature, pH) and establish correlation models linking wettability, roughness, and interfacial durability to guide engineering material selection and protective design [99].

In the actual service environment of civil engineering, nanocomposites must withstand the combined effects of multiple environmental factors [100]. Wet–dry cycles induce volume changes in the polymer matrix, generating repeated stresses at the interface and causing interfacial fatigue damage. Freeze–thaw cycles produce ice crystal growth pressure in the interfacial region, leading to debonding and microcrack propagation. Ultraviolet radiation triggers degradation and cross-linking of polymer chains, altering the chemical structure and mechanical properties of the interfacial zone. Chemical erosion (e.g., acid rain, salt spray, chloride ion permeation) corrodes the surface of nanofillers or the polymer matrix, weakening the interfacial bond strength [101]. The synergistic effects of these environmental factors accelerate degradation processes in the interface region, leading to issues such as interfacial debonding, chemical structural changes, and deterioration of interfacial phase properties. Ultimately, this causes the nano-reinforcement effect to gradually diminish, resulting in “interface failure”.

**Figure 12:**
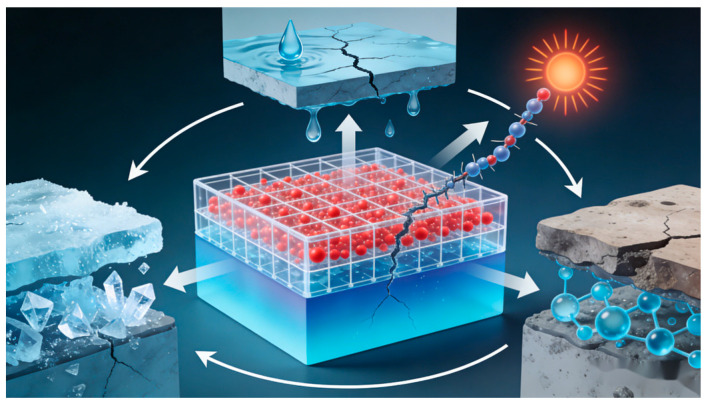
Schematic diagram of the state changes in water molecules in different environments.

The mechanisms of interfacial failure primarily include the following aspects: First, in humid and hot environments, moisture permeates into the interfacial region, weakening secondary bonding interactions such as hydrogen bonds and van der Waals forces, while potentially triggering hydrolysis reactions that lead to chemical bond breakage (as shown in Figure 12). Second, under the coupled effects of mechanical loading and environmental factors, microcracks form in the interfacial region and gradually propagate to form macroscopic damage. Third, the difference in thermal expansion coefficients between nanofillers and the polymer matrix generates thermal stresses at the interface during temperature changes, leading to fatigue damage over time. Fourth, in chemically corrosive environments, corrosive media preferentially attack the interface region, forming corrosion products or altering the chemical composition of the interface, thereby reducing the bond strength [102].

Ensuring the interfacial stability of nanocomposites over decades of service life is the core scientific issue for guaranteeing their long-term performance [103]. This requires in-depth research across multiple levels: At the material design level, novel interface modification techniques must be developed—such as constructing gradient interfaces, introducing reversible bonding, and designing self-healing interfaces—to enhance interface toughness and stability. At the characterization technology level, in situ, real-time interface performance testing methods must be advanced to reveal interface evolution patterns under complex environments. At the theoretical modeling level, establishing multiscale, multiphysics-coupled interface failure prediction models is essential to provide theoretical guidance for material design and service life assessment. At the engineering application level, developing interface-performance-based methods for predicting material service life is crucial to furnish scientific basis for engineering design and maintenance. Only by systematically addressing interface stability issues can the long-term reliable service of nanopolymer composites in civil engineering be ensured, enabling the full realization of their performance advantages [104].

Large-scale manufacturing processes represent a critical engineering hurdle that must be overcome for nanopolymer composites to transition from laboratory research to engineering applications. While laboratory-scale preparation methods such as solution blending and in situ polymerization have yielded significant results in fundamental research, they reveal numerous limitations when confronting the demands of civil engineering for high-volume, continuous, and low-cost material production [105]. The solution blending method requires substantial amounts of organic solvents, posing challenges such as high costs, difficult solvent recovery, and environmental pollution, making it difficult to meet green production requirements. While the in situ polymerization method can achieve good dispersion of nanofillers, it involves harsh reaction conditions and complex processes, often leading to batch-to-batch performance variations during scale-up. Furthermore, laboratory methods typically employ batch operations, resulting in low production efficiency that struggles to meet the large-scale material demands of civil engineering.

To address these challenges, melt blending extrusion has emerged as one of the most promising industrial-scale production technologies [106]. This technology leverages the high shear force field of twin-screw extruders to achieve dispersion and mixing of nanofillers in the molten state, offering distinct advantages such as solvent-free operation, continuous production capability, and high efficiency. However, scaling up presents two core challenges: first, the high-temperature, high-shear environment may damage nanofiller structures or induce polymer degradation; second, the narrow process window requires precise, coordinated control of parameters including screw configuration, temperature, rotational speed, and feeding. Research indicates that technical strategies such as segmented shearing, optimized screw combinations, and lateral feeding can reduce material damage while ensuring dispersion quality [107,108].

**Figure 13:**
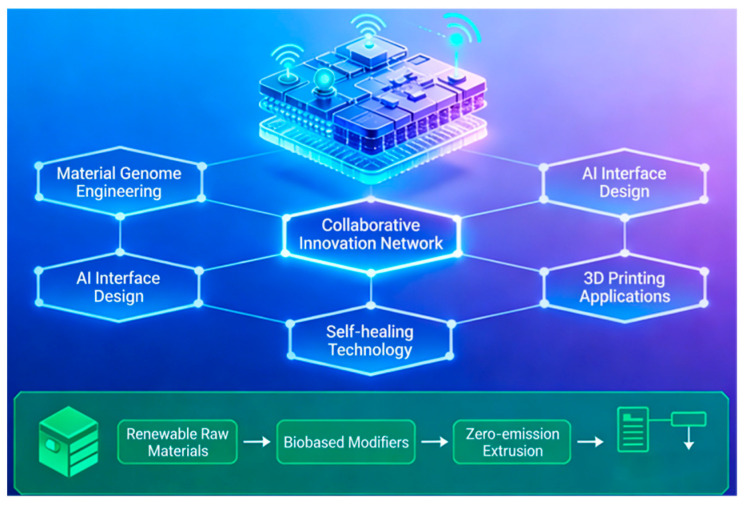
Schematic diagram of collaborative innovation network and green preparation process.

More innovative online modification processes represent the direction of advanced integrated manufacturing [109]. This process simultaneously achieves surface functionalization of nanofillers and melt blending of composites within a single system. By injecting modifiers online, it enables in situ surface modification of nanofillers, not only streamlining production but also enhancing interfacial stability through chemical bonding. The further developed reactive extrusion technology fully integrates polymerization reactions with the compositing process, achieving highly intensive manufacturing. Industrializing these advanced processes requires overcoming several engineering challenges: developing precision feeding systems capable of micro-quantity, continuous, and stable delivery of nanofillers; establishing quantitative models linking process parameters to material structure and properties; constructing intelligent production systems integrating online monitoring and feedback control (as shown in Figure 13); and reducing production costs through equipment innovation and process optimization [110]. Only by systematically addressing the full spectrum of technical challenges from laboratory development to industrial-scale production can we truly advance the widespread application of nanopolymer composites in civil engineering, providing high-performance, cost-effective advanced material solutions for infrastructure construction.

#### 4.1.2. Performance Evaluation Limitations

The existing evaluation system struggles to comprehensively and accurately predict the long-term behavior of nanocomposites in practical engineering applications. This limitation has become a critical bottleneck constraining their engineering implementation. The current evaluation system primarily faces two core issues: a lack of standardization and a scarcity of long-term service data. These problems directly impact the objective assessment of material performance and the reliable prediction of service life.

The lack of standardized testing methods remains the primary obstacle hindering technological exchange and evaluation. Currently, no widely accepted international or industry standards exist for performance testing of nanomodified coatings, sealants, and composite materials—including assessments of nanodispersion, interfacial strength, and specific functionalities such as self-healing efficiency and self-sensing sensitivity [111]. Significant differences exist among research institutions in testing methods, sample preparation techniques, and evaluation criteria systems, resulting in a lack of comparability between research data and hindering objective technical comparisons and performance evaluations. For instance, in characterizing nanoparticle dispersion, some studies employ scanning electron microscopy (SEM) or transmission electron microscopy (TEM) for morphological observation, while others utilize Raman spectroscopy or X-ray diffraction (XRD) for quantitative analysis. The substantial differences in sensitivity and accuracy between these methods make direct comparison of results challenging [112]. In terms of interface strength testing, the absence of standardized testing methods and evaluation criteria has led to significant challenges. Different studies employ varying interface shear strength testing approaches—such as the droplet embedding method and single-fiber pull-out method—along with differing experimental conditions. This inconsistency makes it difficult to compare interface performance data across studies. This lack of standardization not only hinders academic exchange and technological advancement but also increases risks in engineering applications. Designers are unable to select materials and evaluate performance based on unified standards [113].

Long-term field exposure data and reliable lifespan prediction models represent another weak link in the evaluation system [114]. Currently, most research on the weather resistance and durability of nanocomposites relies on short-term accelerated aging tests, such as xenon lamp aging, salt spray testing, and humid heat aging. However, accelerated aging conditions differ significantly from the real, variable, and coupled outdoor environment, leading to considerable uncertainty in the predicted results [115]. Actual service environments often involve the combined effects of multiple environmental factors, whereas accelerated aging tests typically simulate only a single or a few factors, making it difficult to accurately reflect a material’s long-term behavior under complex conditions. More critically, the lack of systematic, long-term field exposure test data and databases makes establishing accurate and reliable service life prediction models exceptionally challenging. Most existing life prediction models rely on empirical formulas or simplified physical models, making it difficult to accurately predict the performance evolution of materials during long-term service. While physical models can describe material degradation mechanisms at the fundamental level, they require extensive experimental data for validation and refinement. Data-driven models, though capable of learning complex nonlinear relationships from data, necessitate sufficient long-term service data as training samples [116]. The lack of long-term field exposure data limits the predictive accuracy and reliability of these models, increasing risks in engineering applications. Therefore, establishing a systematic field exposure testing network to accumulate long-term service data, and developing multiscale, multi-physics-field coupled life prediction models based on this data, is crucial for advancing the safe and reliable application of nanocomposites in civil engineering.

Specifically, the lack of long-term durability data manifests in the following aspects:

Gaps in databases for real-world environmental coupling effects: Existing accelerated aging tests (such as xenon arc aging and salt spray tests) primarily simulate a single or a few dominant factors. However, actual engineering environments are complex systems where multiple factors—temperature, humidity, ultraviolet radiation, chemical corrosion, mechanical loading, and freeze–thaw cycles—interact in spatiotemporal coupling. These factors may exhibit synergistic acceleration of degradation (e.g., UV aging induces microcracks on polymer surfaces, thereby enhancing moisture and ion penetration and accelerating hydrolysis and corrosion) or antagonistic interactions. Currently, publicly available, systematic long-term natural exposure databases for nanocomposites across different climate zones and engineering locations (e.g., coastal splash zones, industrial atmospheric zones, freeze–thaw cycling zones) are virtually nonexistent. This severely limits understanding of their actual service behavior and model calibration.

Lack of in situ monitoring data linking performance degradation to interfacial failure: The core of nanocomposite performance degradation often lies in the failure of the nanofiller–matrix interface. However, the vast majority of long-term studies currently focus solely on endpoint testing of macroscopic properties (such as strength loss, impedance reduction, color change), lacking in situ, non-destructive monitoring data on the chemical state of the interface, morphological evolution, stress redistribution, etc., during the aging process. This “black-box” approach to aging assessment fails to reveal the microscopic mechanisms of performance degradation and cannot provide critical inputs for establishing physics-based life prediction models.

Lack of repair and maintenance cycle data: For smart nanocomposites claiming self-healing capabilities, long-term validation data is lacking regarding the decay patterns of repair efficiency over service time and repair cycles, as well as the reliability of triggering repair conditions in complex environments. This directly undermines the feasibility and credibility of engineering structures’ full lifecycle cost and maintenance planning based on such novel materials.

Therefore, establishing a cross-regional, cross-disciplinary joint exposure testing network and employing advanced in situ characterization techniques for specimen tracking and monitoring is the essential path to accumulating valuable long-term data and overcoming current durability evaluation challenges.

Although laboratory studies have played a crucial role in elucidating the reinforcement mechanisms of nanocomposites and screening material systems, significant discrepancies remain between their results and the long-term behavior of materials in real-world service environments. Laboratory tests are typically conducted under a single or simplified environmental conditions (e.g., constant temperature and humidity, single corrosive medium, fixed-frequency cyclic loading). In contrast, actual engineering structures endure complex interactions involving multi-field coupling, variable-amplitude alternating loads, and spatio-temporal non-uniformity (e.g., coupling of temperature-humidity cycles with vehicle dynamic loads, synergistic effects of freeze–thaw cycles and salt corrosion, interplay between ultraviolet radiation and mechanical fatigue). This discrepancy manifests primarily in the following aspects: ① Difficulty in simulating environmental coupling effects: Accelerated aging tests (e.g., xenon lamp aging, salt spray tests) often fail to replicate the synergistic, antagonistic, or sequential interactions among multiple factors in real environments. For instance, polymer chain scission induced by UV radiation may accelerate hydrolysis in humid-heat conditions, while freeze–thaw cycles may exacerbate the propagation of existing microcracks—coupling mechanisms that are difficult to fully capture in single-factor tests. ② Discrepancies between load spectra and service histories: Laboratory mechanical tests typically employ constant-amplitude or simplified spectral loads, whereas actual structural loads exhibit randomness, non-proportional multiaxiality, and long-term degradation characteristics. The accumulation of interfacial damage and filler–matrix debonding in nanocomposites under complex stress paths are difficult to comprehensively simulate under laboratory conditions. ③ Scale effects and mismatched boundary conditions: Laboratory specimens are small with controlled fabrication processes, whereas actual engineering components are large with high construction variability. This leads to systematic differences in internal defect distribution, residual stresses, and interface states compared to lab specimens, undermining the reliability of performance extrapolation.

Therefore, future efforts should focus on establishing a “laboratory-to-field” evaluation linkage system. This involves setting up long-term exposure test stations, conducting full-scale component coupled environmental-mechanical tests, and developing performance evolution models based on in situ monitoring and digital twins. Gradually building a predictive bridge from laboratory parameters to engineering service life will reduce uncertainties in the large-scale application of nanocomposites in civil engineering.

#### 4.1.3. Economic and Environmental Barriers

Cost and safety factors are key determinants of a technology’s market acceptance. Challenges in these two areas directly impact the commercialization and application prospects of nanocomposites in civil engineering. Cost–benefit analysis represents the primary economic factor influencing technology adoption [117]. The production costs of high-performance nanomaterials remain relatively high, primarily due to their complex synthesis processes, stringent purity requirements, and limitations in large-scale production technologies [118]. Taking carbon nanotubes as an example, their production costs typically range from several hundred to several thousand yuan per kilogram, far exceeding those of traditional filler materials. Furthermore, achieving uniform dispersion of nanofillers within polymer matrices requires complex surface modification techniques and efficient dispersion processes. These processes not only increase equipment investment and energy consumption costs but also extend production cycles. Overall, the initial cost of nanocomposites is generally 30–100% higher than that of traditional materials, or even more. Although nanocomposites may offer potential long-term benefits such as reduced maintenance costs and extended service life, a clear, full-lifecycle cost–benefit analysis model remains underdeveloped. Such analysis requires comprehensive consideration of multiple factors including material initial cost, construction cost, maintenance cost, benefits from extended service life, potential failure risks, and environmental impact. Currently, research on full life-cycle cost analysis for different application scenarios remains relatively scarce, lacking standardized evaluation methods and database support. This uncertainty makes owners and contractors more cautious in decision-making, leading them to favor traditional materials with clear costs and high technical maturity. Consequently, this affects the market acceptance of nanocomposites.

Environmental and health risks (EHS) associated with nanomaterials represent non-technical societal issues that must be addressed during technology deployment. Nanomaterials may pose release risks during production and construction processes, particularly during operations such as spraying, cutting, and grinding, where nanoparticles can enter the human body through inhalation, skin contact, or ingestion. Research indicates that certain nanomaterials may induce biological effects such as inflammatory responses, oxidative stress, and cytotoxicity upon entering the body. Long-term exposure may pose potential hazards to the respiratory and cardiovascular systems. Furthermore, the migration, transformation, and fate of nanomaterials in the environment remain incompletely understood, and their long-term impacts on ecosystems require further investigation.

Currently, research on the environmental, health, and safety (EHS) aspects of nanomaterials remains in the assessment phase, lacking a systematic risk assessment framework and standardized testing methods. Significant variations exist in the toxicity, bioavailability, and environmental behavior of different nanomaterials, necessitating tailored assessments for specific materials and application scenarios. Establishing safe production, construction, usage, and disposal protocols is an urgent priority. This includes workplace exposure limits, personal protective equipment requirements, and waste management guidelines. Simultaneously, addressing public safety concerns about “nano” technologies is crucial for their widespread adoption. Building public trust and acceptance requires science communication, transparency, and public engagement.

In summary, cost–benefit analysis and environmental health and safety concerns represent critical barriers that must be addressed for the widespread adoption of nanocomposites in civil engineering. Only through technological innovation to reduce production costs, the establishment of comprehensive life-cycle cost analysis models, the formulation of scientific safety regulations and standards, and enhanced public communication and risk management can nanocomposites transition from laboratory research to engineering practice, enabling their large-scale application in infrastructure construction.

#### 4.1.4. The Scalability Gap from Laboratory to Construction Site

Although laboratory studies have demonstrated the exceptional performance of nanopolymer composites, one of the core obstacles to their transition toward large-scale engineering applications is the formidable challenge of scalability. This extends far beyond mere production scale-up, encompassing the adaptability of the entire chain—from material preparation and construction techniques to quality assurance.

Consistency and Stability in Material Preparation: Laboratory methods like solution blending and in situ polymerization achieve excellent filler dispersion and interface control in small batches. However, these techniques typically rely on substantial solvents, precise process control, or specific reaction conditions—making them incompatible with civil engineering’s demand for low-cost, high-efficiency, continuous production. During scale-up, issues such as batch-to-batch dispersion stability of nanofillers, storage stability of prepregs or masterbatches, and polymer degradation caused by shear heat during large-scale mixing become prominent, directly impacting the uniformity and reliability of final product performance.

Adaptability to field application processes: Nanomodified materials (particularly coatings and sealants) are often more sensitive to application environments (temperature, humidity, substrate cleanliness) and techniques (mixing ratios, stirring methods, coating/injection technologies, curing conditions). For instance, nanofillers may settle or re-agglomerate in two-component field-mixed systems due to inadequate mixing or mismatched gelation times; nanomodified epoxy structural adhesives may exhibit lower tolerance for damp substrates. Currently, the absence of standardized field application guidelines and quality control checkpoints tailored to nanomaterial characteristics increases construction quality uncertainty.

Mismatched performance verification scales: Laboratory performance tests rely on small standard specimens, whose stress states, defect distributions, and environmental exposure uniformity fundamentally differ from actual large-scale engineering components (e.g., tens-of-meters-long FRP bars, hundreds-of-square-meter coating areas). Size effects and boundary effects make it difficult to directly extrapolate the superior performance of small specimens to large structures. For example, the excellent crack resistance of coatings on small test panels may fail when confronted with macro-cracks in large concrete structures caused by thermal shrinkage; the high bond strength of FRP bars under short anchorage lengths may not be fully realized in long-span components due to uneven stress distribution.

Therefore, bridging the scalability gap requires close collaboration between academia and industry. This partnership should focus on developing engineering-ready fabrication processes, establishing performance-based on-site construction and acceptance standards, and conducting long-term performance validation tests on full-scale or large-scale components. Such efforts will help close the knowledge gap between “sample performance,” “component performance,” and ultimately “structural performance.”

#### 4.1.5. The Challenge of Balancing Cost, Performance, and Environmental Benefits

The market acceptance of nanopolymer composites ultimately hinges on whether they can achieve a superior overall balance compared to traditional materials across three dimensions: cost, performance enhancement, and environmental impact. Currently, this field faces a dual challenge of “cost-effectiveness” versus “environmental sustainability.”

High initial costs and ambiguous lifecycle benefits: Cost structure: Total costs encompass not only the premium price of nanofillers themselves (e.g., high-purity carbon nanotubes, functionalized graphene) but also manufacturing expenses incurred from surface modifiers, specialized dispersion equipment, and additional processing steps (such as ultrasonic treatment) required for uniform dispersion and stable interfaces. This typically elevates the initial cost of nanocomposites by 30% to several times that of conventional materials. Absence and uncertainty in life cycle cost analysis (LCCA): While nanomaterials theoretically offset initial costs by extending maintenance intervals, reducing servicing frequency, and prolonging structural lifespan, conducting reliable LCCA faces significant challenges: (a) Insufficient long-term performance data, as previously noted, renders precise predictions of “lifespan extension” highly uncertain. (b) Estimating maintenance costs is difficult, as repairs for nanomaterial-modified structures may require specialized materials or processes with unknown expenses. (c) Quantifying failure risk costs is challenging. Without sufficient validation data and proven application cases, owners and engineers often favor traditional solutions with clear initial costs and high technical maturity to avoid potential technical and economic risks.

Full-chain management challenges for environmental, health, and safety (EHS) risks: The “Green Paradox”: While some nanomodifications aim to enhance durability for resource conservation, their production processes (e.g., energy-intensive synthesis of nanofillers, use of organic solvents) and end-of-life stages (e.g., recyclability challenges for composites containing inseparable nanofillers) may impose new environmental burdens. For instance, certain nanofunctional coatings may shed nanoparticles into surrounding soil and water environments during end-of-service due to aging, with their long-term ecological toxicity not yet fully understood. Occupational exposure and construction safety: Engineering-grade nanoparticles (distinct from highly agglomerated product forms) may be released during material production, on-site cutting, grinding, spraying, and other processes, posing occupational exposure risks. Currently, monitoring standards, safety protocols, and exposure limits for nanomaterial exposure at civil engineering construction sites remain inadequate, increasing health risks for workers and management costs. Public Perception and Acceptance: Public concerns about the potential risks of “nanotechnology” (though possibly exaggerated) may translate into resistance toward public infrastructure using such materials, creating barriers to societal acceptance.

Therefore, future development must adopt a more comprehensive and transparent assessment framework. This includes developing low-cost, low-environmental-impact synthesis and modification technologies for nanofillers; establishing and publicly disclosing cradle-to-grave life cycle assessment (LCA) and life cycle cost analysis (LCCA) data; and formulating EHS management specifications and standards covering production, transportation, construction, use, and disposal phases. Only when new materials demonstrate clear and verifiable comprehensive advantages across technical performance, economic cost, and environmental–social impacts will they achieve widespread adoption in the highly competitive civil engineering materials market.

### 4.2. Future Research Directions

The preceding analysis of challenges reveals that the key to advancing nanopolymer composites toward engineering applications lies in overcoming systemic bottlenecks during the transition from “exceptional samples” to “reliable engineering products.” Future research must not only focus on material innovation but also prioritize establishing a comprehensive technological framework and application ecosystem that supports scalable manufacturing, predictable performance, and integrated evaluation. To address the aforementioned challenges, future research should focus on the following cutting-edge directions to advance the intelligent, sustainable, and engineering-oriented development of nanopolymer composites in civil engineering.

#### 4.2.1. Material Design Innovation

Self-healing nanocomposites: future research will go beyond single reinforcement or protective functions, focusing on designing smart composites with dynamic response capabilities.

Embedding nano-capsules encapsulating repair agents or nano-carriers featuring reversible chemical bonds (such as Diels–Alder bonds or hydrogen bonds) into a polymer matrix: When micro-cracks form in the material, the nano-carriers rupture or undergo chemical bond rearrangement upon external stimuli (heat, light), enabling autonomous repair at the damaged site. This significantly enhances the durability of concrete structures, coatings, and sealants.

Self-sensing nanocomposites: Leveraging the exceptional conductivity of carbon-based nanomaterials (carbon nanotubes, graphene) and their resistance changes under strain or damage, or by integrating fluorescent nanoparticles, coatings and composites with self-sensing capabilities for stress/strain, damage, and corrosive ion permeation are developed. These materials serve as “nerve sensors” for structural health monitoring, enabling real-time, distributed sensing of infrastructure conditions.

#### 4.2.2. Sustainable Development

Reducing environmental footprints is an inevitable requirement for future material development. Utilizing bio-based nanomaterials—such as nanocellulose (CNC, CNF)—with their exceptional properties, including high strength, high modulus, renewability, and biodegradability, presents immense potential as green nanofillers. Research into their dispersion within polymer matrices, interfacial regulation, and their enhancing effects on the mechanical and barrier properties of composites can lead to the development of high-performance, eco-friendly civil engineering materials. Nanoscale utilization of industrial byproducts: Industrial byproducts such as fly ash, slag, and silica fume can be processed at the nanoscale or have their nano-sized active components extracted for use as low-cost nanofillers or reactive constituents. This approach not only enhances composite properties (e.g., improving density and reducing shrinkage) but also enables high-value utilization of waste, aligning with circular economy principles.

#### 4.2.3. Integration of Interdisciplinary Approaches

Modern computing and information technology will profoundly empower material research and development, providing revolutionary tools for the design, optimization, and performance prediction of nanopolymer composites. Through the integrated application of advanced computational methods such as multiscale simulation and machine learning, materials R&D is transitioning from the traditional trial-and-error approach to an intelligent paradigm of “design-predict-validate.” This shift holds promise for significantly shortening development cycles, reducing experimental costs, and enabling precise control over material properties.

Multiscale simulation serves as a critical computational tool for understanding the complex behavior of nanocomposites. This approach employs a bottom-up modeling strategy to reveal structure-property relationships across different spatial and temporal scales. At the molecular scale, molecular dynamics simulations precisely describe interfacial interactions, interfacial energy, bonding strength, and load transfer mechanisms between nanoparticle fillers and polymer chains, providing an atomic-level physical picture for understanding interfacial reinforcement effects. At the mesoscale, mesoscopic mechanical models predict the distribution and orientation of nanofillers within the matrix, along with the resulting effective properties of the composite. This establishes quantitative relationships between filler content, dispersion state, and macroscopic performance. At the macroscale, finite element analysis simulates the mechanical response, damage evolution, and failure behavior of composite components or structures under actual service conditions, providing a basis for engineering design and life assessment. Establishing this multiscale simulation framework not only reveals the reinforcement mechanisms and failure modes of nanocomposites in depth but also guides material design. For instance, it enables targeted performance regulation by optimizing interfacial modification strategies, filler morphology, and distribution. More importantly, multiscale simulation reduces reliance on extensive experimentation. Through computational screening, it rapidly eliminates unpromising material designs, concentrating experimental resources on the most promising candidates and significantly enhancing R&D efficiency.

Machine learning is driving a data-driven intelligent revolution in material research and development. This approach leverages high-throughput data generated from existing experiments and simulations, using machine learning algorithms to uncover complex nonlinear relationships between composition, processing, structure, and properties. In material screening, machine learning can rapidly evaluate thousands of potential nanofiller/matrix combinations, predict their performance, identify the most promising candidate materials, and avoid blind experimentation. For performance prediction, trained models can accurately forecast material properties under unknown formulations or processing conditions, providing reliable guidance for new material development. More revolutionary is the reverse-engineering capability: given target performance requirements, machine learning models can deduce the material composition and preparation process needed to meet these specifications, enabling “on-demand design” for customized materials. This data-driven design paradigm, combined with high-throughput computation and experimental validation, is establishing a complete closed-loop material R&D system, significantly shortening the cycle from material discovery to engineering application. For instance, machine learning models can rapidly screen surface modification schemes for nanofillers with optimal interfacial performance or predict the long-term durability evolution of materials under different service environments, providing scientific basis for civil engineering applications.

The integration of multiscale simulation and machine learning represents the future direction of material research and development. On one hand, multiscale simulation can generate vast amounts of high-quality “virtual data,” compensating for experimental data gaps and providing rich training samples for machine learning models. On the other hand, machine learning can accelerate multiscale simulation processes—for instance, by replacing computationally expensive molecular dynamics simulations with surrogate models to enable rapid performance prediction. This dual-engine approach of “computing + data” is propelling materials R&D from experience-driven methods toward theory-guided, data-driven intelligent development, providing robust support for innovative applications of nanopolymer composites in civil engineering.

#### 4.2.4. Standardization and Regulation Development

Advancing technological standardization is a critical step toward industrialization. Promoting the conversion of standards and guidelines: Academia, industry, and standard-setting bodies must collaborate closely to jointly establish a series of standards, technical specifications, and design guidelines for evaluating performance, designing, constructing, and accepting nanopolymer composite materials in civil engineering. This encompasses material classification standards, durability testing standards, construction process regulations, and performance-based design methodologies. Only through establishing a comprehensive standards system can designers, engineers, and regulatory authorities be provided with clear guidelines, thereby lowering application barriers and risks. This ultimately enables the safe, reliable, and large-scale engineering application of this technology.

**Figure 14:**
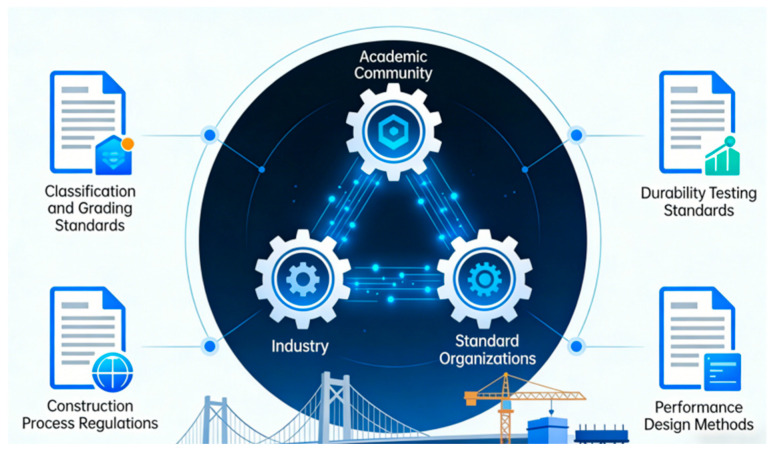
Schematic diagram of collaborative innovation in engineering construction standards.

Conclusion: Nanopolymer composites present revolutionary opportunities for enhancing the performance, durability, and intelligent capabilities of civil engineering infrastructure. Despite persistent challenges in technology, evaluation, and sustainability, focusing on future directions—including intelligent material design, green sustainable pathways, interdisciplinary R&D tools, and standardized systems—holds promise for systematically overcoming current bottlenecks. This will ultimately propel these advanced materials from research into widespread engineering practice, laying the material foundation for constructing safer, more durable, smarter, and more sustainable future infrastructure (as shown in Figure 14).

## 5. Conclusions

This paper systematically reviews the core scientific mechanisms, application outcomes, existing challenges, and future directions of nanopolymer composites in enhancing material weather resistance and toughness within civil engineering. Nanofillers such as carbon nanotubes, graphene, and nano-silica form physical adsorption (van der Waals forces, hydrogen bonds) and chemical bonding (covalent bonds, ionic bonds) with polymer matrices to create highly efficient stress transfer and energy dissipation interfaces. This synergistically enhances the materials’ resistance to UV aging, corrosion, and freeze–thaw cycles. Simultaneously, mechanisms such as crack deflection, bridging, pinning, and matrix shear yielding significantly improve the materials’ toughness, strength, fatigue resistance, and impact performance. In critical applications such as protective coatings, sealing and bonding materials, and composite structural components, nanopolymer composites demonstrate outstanding performance advantages. They effectively address the shortcomings of traditional materials—including insufficient durability, poor toughness, and limited functionality—offering immense potential for extending infrastructure service life, reducing maintenance costs, and enabling lightweight and intelligent structures. For instance, graphene-modified anti-corrosion coatings substantially enhance the barrier protection of steel structures, while nanomodified epoxy adhesives significantly improve the bond strength at FRP–concrete interfaces. Nevertheless, the large-scale engineering application of nanopolymer composites still faces multiple severe challenges. Technologically, uniform dispersion of nanofillers, long-term interfacial stability, and large-scale manufacturing processes remain critical bottlenecks requiring breakthroughs. In performance evaluation, the absence of standardized testing methods and limited long-term field exposure data compromise the accuracy of service life predictions. Economically and environmentally, high production costs and potential environmental health risks constrain market adoption.

To advance the industrialization and sustainable development of nanopolymer composites in civil engineering, future research should focus on four key directions: first, developing multifunctional intelligent materials with self-healing and self-sensing capabilities to meet the growing functional demands of infrastructure; second, implementing green circular economy principles by utilizing bio-based nanomaterials and industrial byproducts to reduce environmental footprints; third, integrating interdisciplinary approaches such as multiscale simulation and machine learning to achieve intelligent and efficient material development; and fourth, establishing comprehensive standards for performance evaluation, design, construction, and acceptance to provide reliable foundations for engineering applications.

In summary, nanopolymer composites present revolutionary opportunities for upgrading and innovating civil engineering materials. It is particularly noteworthy that the dispersion quality of nanofillers exerts a controlling influence on their final performance that is no less significant than, and may even surpass, the effects of filler type and dosage. Future research should place greater emphasis on standardizing dispersion processes, developing online monitoring and control technologies, and establishing “dispersion structure-property mapping” models using machine learning methods. This approach will reduce performance variability and enhance the applicability and reliability of composite materials in civil engineering. Simultaneously, for nanocomposites to transition from laboratory research to engineering applications, they must bridge the gap between “laboratory performance” and “long-term field behavior.” Future research should prioritize systematic evaluations under conditions approximating real-world service environments—such as multi-field coupling, variable-amplitude loading, and large-scale components. By integrating structural health monitoring data with machine learning methodologies, we can develop cross-scale predictive models spanning material composition to structural lifespan. This approach will provide the scientific foundation for reliable deployment of nanocomposites in critical infrastructure. By overcoming core challenges in technology, evaluation, economics, and environmental sustainability, and continuously advancing innovations in material design, preparation processes, and application technologies, nanopolymer composites will undoubtedly find widespread application in engineering practice. This will lay a solid foundation for constructing safer, more durable, smarter, and more sustainable modern civil engineering infrastructure.

## Figures and Tables

**Table 1:** Comparative analysis of this review and recent related reviews.

Comparison Dimensions	This Work	Wei et al. [10]	Liu et al. [9]	General Reviews
Core Perspective	Optimization of Weather Resistance and Toughness in Nanopolymer Composites for Civil Engineering Applications	Preparation and Properties of Epoxy/Graphene Nanocomposites	Application of Nanomaterials in Ultra-High Performance Concrete	Cross-Domain Applications of Polymer Nanocomposites
Material System	Polymer matrix (epoxy/polyurethane/vinyl ester, etc.)+nanofillers (0D/1D/2D)	Epoxy+Graphene (Single System)	Cement matrix+nanomaterials	Multiple matrixes+multiple fillers
Theoretical Framework	Structure-Property-Application Integration Framework Interface Engineering Center Theory	Dispersion and Interface Engineering	Mechanism of Nanomaterials in Cement Hydration	Material Preparation and Performance Characterization
Performance Focus	Weather resistance (UV/corrosion/freeze–thaw)+Toughness (impact-resistant/reinforced/dynamic load)	Mechanical+Electrical+Thermal+Flame Retardant Properties	Mechanical strength+Durability+Workability	Overview of Multifunctional Performance
Application Integration	Three Major Application Areas Integrated: Protective Coatings/Sealing and Bonding Materials/Composite Structural Components	General Industrial Applications	Concrete Structural Engineering	Cross-Domain Application Overview
Mechanism Depth	Multiscale Mechanism Correlation: Molecular Interface Interactions → Macroscopic Properties	Multiscale Interfacial Coupling and Dispersion Mechanisms	Volcanic ash/filling/nucleation effect	Single Mechanism Description
Future-oriented	Smart Materials+Green Sustainability+Computation-Driven+Standardization	Industrial Application Development	Development of High-Performance Concrete	General Future Trends

**Table 2:** Summary of modification mechanisms, targeted properties, and application fields for nanoparticle fillers of different dimensions.

Dimension	Representative Filler	Core Interface Mechanisms and Modification Strategies	Key Performance Improvements (Goals)	Typical Application Areas (See Section)
0D (nanoparticles)	Nano-SiO_2_, TiO_2_, ZnO, rubber particles	Physical adsorption/chemical grafting: Improving compatibility and dispersion through silane coupling agents (e.g., PUCA [38]) or polymer grafting. Functional mechanisms: UV absorption/scattering, crack pinning, inducing matrix shear yielding, pore filling.	Weather resistance: Resists UV aging and increases glass transition temperature (Tg). Mechanical properties: Enhances hardness and modulus while significantly improving toughness (impact resistance).	Protective coatings (Section 3.1), Sealants (Section 3.2.1), Cementitious composites (Section 3.3.2)
1D (Nanofibers/Tubes)	Carbon nanotubes (CNTs)	Chemical bonding/mechanical interlocking: Surface carboxylation, amination, or covalent linkage to polymer chains. Functional mechanism: Crack bridging and fiber pull-out, formation of conductive/thermal networks, three-dimensional network reinforcement.	Mechanical properties: Significantly enhanced fracture toughness, fatigue strength, and impact resistance. Functionality: Conductive, thermally conductive, and self-sensing.	FRP composite structural components (Section 3.3.1), High-strength toughness composites, smart coatings
2D (Nanosheets)	Graphene, Graphene Oxide (GO), Nanoclay	Large-area interfacial bonding/physical barrier: Covalent/non-covalent functionalization (e.g., QA bridging [42]), interlayer intercalation. Functional mechanisms: “Maze” barrier effect, crack deflection, interfacial reinforcement, electrochemical protection.	Weather resistance: Exceptional water barrier, oxygen barrier, and corrosion resistance. Mechanical properties: Simultaneously enhances strength, modulus, and toughness.	High-performance anti-corrosion coatings (Section 3.1.1), high-strength composite materials and adhesives (Section 3.2.2 and Section 3.3.1)

**Table 3:** Typical systems and performance gains of nanomodified protective coatings.

Coating Type and Target	Key Nanoparticle Fillers	Typical Addition Amount (wt.%)	Core Performance Gains	Key Influencing Factors/Strategies
Corrosion Protection for Steel Structures	Functionalized Graphene	0.1–1.5	Frequency impedance modulus increased by 1–3 orders of magnitude (e.g., >10^10^ Ω·cm^2^) [38] Salt spray protection time extended by 200–300% [69]	Filler dispersion, layer orientation, and interfacial bonding with epoxy (covalent modification)
	Three-dimensional porous graphene	~0.1	Corrosion current density reduced by an order of magnitude [69]	Three-dimensional interconnected structures promote synergistic interaction between zinc powder conductive networks and physical barriers.
Concrete Surface Protection	Nano-SiO_2_	1.0–2.0	Concrete carbonation depth reduced by 43–44% [71] Projected coating lifespan extended by 50–80% [71]	Fill coating micro-defects to enhance density; control dosage to prevent increased brittleness.
	Nano TiO_2_ (modified)	~1.5	Significantly delays asphalt UV aging, with the carbonyl index decreasing from 5.16 to 0.28 [73]	Synergizes with montmorillonite (MMT) to form a network that provides UV shielding and oxygen barrier properties.
Self-cleaning/Antibacterial	Nano TiO_2_	1.0–5.0	Photocatalytic degradation of organic matter, superhydrophilic (contact angle <5°) Antibacterial rate >99% (with nano-Ag/ZnO composite)	Crystal structure, specific surface area, dispersibility; long-term photocatalytic stability must be considered.
	Nano-SiO_2_/Fluoride	Variable	Fabrication of superhydrophobic surfaces (contact angle >150°, rolling angle <10°) [50]	Micro-Nano Rough Structure Fabrication and Low Surface Energy Material Modification

**Table 4:** Quantitative examples of enhanced key mechanical properties in nanoscale-modified composites.

Matrix Material	Nanofillers and Modification Methods	Addition Amount (wt.%)	Performance Test Metrics	Performance Gain (%)	Core Mechanism (see Section)
Epoxy resin	QA Covalent–Noncovalent Bridge-Modified Graphene [42]	0.2	Tensile strength	+270 (from 5.64 MPa to 20.88 MPa)	Interface reinforcement, efficient stress transfer (Section 2.3.2)
	Amino-oligoimide grafted GO [19]	Not specified	Fracture toughness (KIC)	+56	Crack bridging and interfacial covalent bond disruption energy dissipation (Section 2.3.1)
	CNT-GNP Hybrid Filler [84]	Variable	Modulus/strength, with self-sensing capability	Significantly enhanced, achieving linear resistance changes under strain	Synergistically dispersed to form a three-dimensional conductive and reinforcing network
Carbon Fiber Reinforced Polymer (CFRP)	Gelatin-modified carbon nanotube (g-CNT) fiber interfacial reinforcement [85]	Gelatin-modified carbon nanotube (g-CNT) fiber interfacial reinforcement [85]	Interface normal strength (IFNS)	+40.3	Improving fiber surface roughness and chemical activity (Section 2.1)
	Gelatin-modified carbon nanotube (g-CNT) fiber interfacial reinforcement [85]	0.1	Bending strength	+20.3	Enhancing the properties of the matrix itself and interfacial bonding (Section 2.3.2)
Cementitious materials	Synergistic effect of nano-SiO_2_ with steel-PVA fibers [86,88]	~3.0	Compressive/flexural strength, bond strength	Significant improvement, with porosity reduced by ~37% [89]	Pore filling and microstructure densification, hydration promotion, fiber–matrix interface optimization (Section 2.2.3 and Section 2.3.1)

**Table 5:** Comprehensive evaluation of engineering applications for major nanoparticle filler systems.

Nanofiller System	Core Strengths (Drivers)	Primary Drawbacks/Challenges (Obstacles)	Most Suitable Scenarios	Key Bottlenecks for Large-Scale Application
Carbon-based materials (CNTs, graphene)	Superior mechanical properties (strength and toughness) Excellent electrical/thermal conductivity High barrier properties (2D materials)	High cost Difficult to disperse and control at the interface Insufficient long-term environmental stability data (e.g., oxidation) Potential EHS risks (especially for CNTs)	High-performance corrosion protection, structural reinforcement, smart sensing and monitoring	Low-cost mass production, stable dispersion process, reliable long-life evaluation
Metal oxides (Nano-SiO_2_,TiO_2_, ZnO)	Relatively low cost Well-defined functionality (UV shielding, photocatalysis, filling) Easily modifiable surface properties	Prone to agglomeration, requiring surface treatment High loading levels may compromise matrix toughness Photocatalytic activity of certain materials (e.g., nano-TiO_2_) may degrade organic matrices	Concrete modification, functional coatings (self-cleaning, UV-resistant), sealant reinforcement	Balancing functionality and durability, long-term performance validation in complex environments
Nano-clay/minerals (Montmorillonite, illite, etc.)	Low cost and wide availability Excellent barrier and reinforcing properties Good thermal stability and flame retardancy	Difficult interlayer separation, limiting efficiency enhancement May affect substrate transparency and processing fluidity	Flame-retardant composite materials, medium-to-low requirement anti-corrosion coatings, green building materials	High-efficiency, eco-friendly stripping and modification technology with uniform performance control
Bio-based/waste-derived nanomaterials (nano-cellulose, modified fly ash, etc.)	Environmentally friendly and sustainable Low toxicity or non-toxic Some exhibit excellent mechanical properties	Significant performance fluctuations (source-dependent) Strong hydrophilicity, poor compatibility with hydrophobic polymers Immature industrial extraction and purification technologies	Green building materials, internal curing agents, eco-friendly packaging materials	Performance stability and standardization, low-cost purification processes, and compatibility with traditional processes

## Data Availability

No new data were created or analyzed in this study. Data sharing is not applicable to this article.
